# Effects of Nanopillar Size and Spacing on Mechanical Perturbation and Bactericidal Killing Efficiency

**DOI:** 10.3390/nano11102472

**Published:** 2021-09-22

**Authors:** Amar Velic, Alka Jaggessar, Tuquabo Tesfamichael, Zhiyong Li, Prasad K. D. V. Yarlagadda

**Affiliations:** School of Mechanical, Medical and Process Engineering, Engineering Faculty, and Centre for Biomedical Technologies, Queensland University of Technology, 2 George St, Brisbane, QLD 4000, Australia; a2.velic@qut.edu.au (A.V.); alka.jaggessar@qut.edu.au (A.J.); t.tesfamichael@qut.edu.au (T.T.); zhiyong.li@qut.edu.au (Z.L.)

**Keywords:** nanopatterned surfaces, bactericidal efficiency, biophysical modelling, Helfrich–Canham

## Abstract

Nanopatterned surfaces administer antibacterial activity through contact-induced mechanical stresses and strains, which can be modulated by changing the nanopattern’s radius, spacing and height. However, due to conflicting recommendations throughout the theoretical literature with poor agreement to reported experimental trends, it remains unclear whether these key dimensions—particularly radius and spacing—should be increased or decreased to maximize bactericidal efficiency. It is shown here that a potential failure of biophysical models lies in neglecting any out-of-plane effects of nanopattern contact. To highlight this, stresses induced by a nanopattern were studied via an analytical model based on minimization of strain and adhesion energy. The in-plane (areal) and out-of-plane (contact pressure) stresses at equilibrium were derived, as well as a combined stress (von Mises), which comprises both. Contour plots were produced to illustrate which nanopatterns elicited the highest stresses over all combinations of tip radius between 0 and 100 nm and center spacing between 0 and 200 nm. Considering both the in-plane and out-of-plane stresses drastically transformed the contour plots from those when only in-plane stress was evaluated, clearly favoring small tipped, tightly packed nanopatterns. In addition, the effect of changes to radius and spacing in terms of the combined stress showed the best qualitative agreement with previous reported trends in killing efficiency. Together, the results affirm that the killing efficiency of a nanopattern can be maximized by simultaneous reduction in tip radius and increase in nanopattern packing ratio (i.e., radius/spacing). These findings provide a guide for the design of highly bactericidal nanopatterned surfaces.

## 1. Introduction

Nanopatterning is the process of physically modifying a surface to impart bacteria killing properties to otherwise innocuous materials. It can be performed by various bottom-up or top-down techniques, but principally involves creating an array of nondevelopable nanoscale protrusion (termed ‘nanopillars’) with dimensions of radius, spacing and height usually all on the order of 100 nm or less [1,2]. The idea derives from mimicking nanopatterned insect wings, such as the *Psaltoda claripennis* cicada [3]. The bactericidal efficacy of nanopatterning is not necessarily restricted to any one material or any one cell species. Nanopatterned surfaces of polymer [4,5], metal [6,7,8,9], and metalloid [10,11] material have all been demonstrated to kill several species of both gram-negative and gram-positive bacteria—in some cases even with antimicrobial resistant strains [8,9]. The ability of nanopatterning to confer a broad-spectrum killing effect on diverse materials has implied that the resulting surfaces operate on a mechanism that is predominantly physical. This is most directly inferred from electron micrographs of bacteria adhered to nanopatterned surfaces, which often show instances of nanopillar penetration or piercing [12,13,14,15,16]. Accordingly, it is understood that nanopatterned surfaces kill bacteria by inducing—through contact—mechanical stress and strain in the envelope which exceeds a survivable limit [12,17]. Whether death is the result of only rupture, or also other mechanosensitive physiological effects remains a topic of ongoing study [13,18].

Consistent with this mechanism, the killing efficiency of nanopatterned surfaces is known to be size sensitive; however, a clear design strategy remains elusive. Several experimental studies have established that killing efficiency can be modulated by only varying nanopattern dimensions, such as nanopillar radius, spacing and height [4,5,10,11,14,16,19,20,21,22,23]. Such studies have been typically conducted by evaluating the killing efficiencies of different nanopattern designs from, either, (i) different cicada wing species [19,21,23], (ii) different exposure times with reactive ion etching [10,11,14], or (iii) different write patterns with electron beam lithography [16]. These methods have unique pitfalls. The first two (i and ii) are susceptible to clustered variation—that is, no two cicada species vary in only one of radius, spacing or height, and etching time simultaneously affects the dimensions of all three. The third (iii) suffers from low throughput, meaning that only a few nanopattern design can be feasibly studied, and killing efficiency of the small patterned areas (e.g., 20 × 20 µm^2^ [16]) cannot be evaluated by conventional live/dead staining assays which require larger fields of view. As a result, no experimental studies have been able to demonstrate the killing efficiency for significant combinations of radius, spacing and height sizes, which may therefore be overlooking certain critical combinations. That being said, an overriding theme from the experimental literature on size-modulated nanopattern killing efficiency is that smaller radii and tighter spacings tend to produce increased killing efficiency. More accurately, in almost all such studies, the highest killing efficiency has corresponded to the nanopattern which has both the smallest tip radius and smallest center spacing of those investigated [11,16,19,20,21,22,23].

To provide more comprehensive and detailed insight, biophysical models have been used to study the effects of nanopattern dimensions on stress and strain induced to the bacterial envelope. These models rely on the understanding that increasing envelope stress and strain should increase killing efficiency. To this end, two perfectly contradicting strategies to enhanced killing efficiency have been recommended from theoretical studies: increasing radius and decreasing spacing, as found in Li [24], Li and Chen [25], and Xiao, et al. [26]; and, decreasing radius and increasing spacing, as found in Xue, et al. [27], Mirzaali, et al. [28] and Maleki, et al. [29]. A source of the discrepancy between these models—and therefore their recommended design strategy—is the loading condition applied to the cell. The first set of models consider intermolecular forces between the cell and the nanopattern surface, approximated using an adhesion energy density, whilst the second set use surface-independent loads such as self-weight or the weight of the fluid column above the cell. Bacteria-surface interaction is indeed dominated, below about 50 nm of separation, by intermolecular forces such as London-van der Waals, electric double layer, and acid-base forces [30], and these also exceed any influence of gravity (see Velic, et al. [12] and its supporting materials); hence only the first approach is a valid representation. That being said, one will notice that neither of the two theoretical design strategies seem consistent with the aforementioned experimental trends on killing efficiency, which typically point toward smaller radii and smaller spacings being most bactericidal. For those using surface-independent loads, such as Xue, et al. [27], Mirzaali, et al. [28] and Maleki, et al. [29], the error seems to stem from the loading condition. However, for the intermolecular force-based models, such as Li [24], Li and Chen [25], and Xiao, et al. [26], a more subtle influence may be at play. These studies have based their recommended design strategy exclusively on in-plane effects—specifically, averaged areal strain—which is enhanced as the nanopillar radius and spacing are increased and decreased, respectively. However, in-plane normal stresses and strains alone cannot possibly represent the total stress state of an envelope perturbed by a nanopattern. One could also reasonably expect, amongst other stresses, contact pressures to be experienced by the envelope. Moreover, such out-of-plane effects are likely to be significant, based on the simple fact that the nanopillars are, characteristically, sharp (those which are most highly bactericidal have been reported to contain nanopillar tip diameters between 10 and 100 nm [31] or tip radii between 5 and 50 nm). How the nanopattern dimensions of radius, spacing and height affect other stresses has been mostly ignored.

It was, therefore, the aim of the present work to provide a more holistic view on the effects of nanopattern dimensions on the envelope stress state and concomitant killing efficiency. This was demonstrated using idealized models of the envelope and intermolecular forces (i.e., Helfrich–Canham model, and thermodynamic adhesion energy density, respectively) which could be equilibrium solved analytically, as previously [24,25,26]. However, in addition to the in-plane areal stress, an expression for the out-of-plane contact pressure was derived, based on the tension in the envelope and the curvature of the nanopillar. Both were furthermore incorporated into a combined stress, based on von Mises theory, to represent the total stress state of the envelope. Contour plots were developed to illustrate the effect of any combination of the tip radius and center spacing between 0 and 100 nm and 0 and 200 nm, respectively. The results showed that nanopillar radius also played a critical role in controlling contact pressure, and both contact pressure and areal stress could be enhanced using small-tipped radii with tight packing (i.e., larger *r*/*s*). Trends from previous experimental studies were also investigated to confirm that this design strategy yielded increased killing efficiency.

## 2. Materials and Methods

### 2.1. Geometry and Properties of a Nanopillar

**Figure 1:**
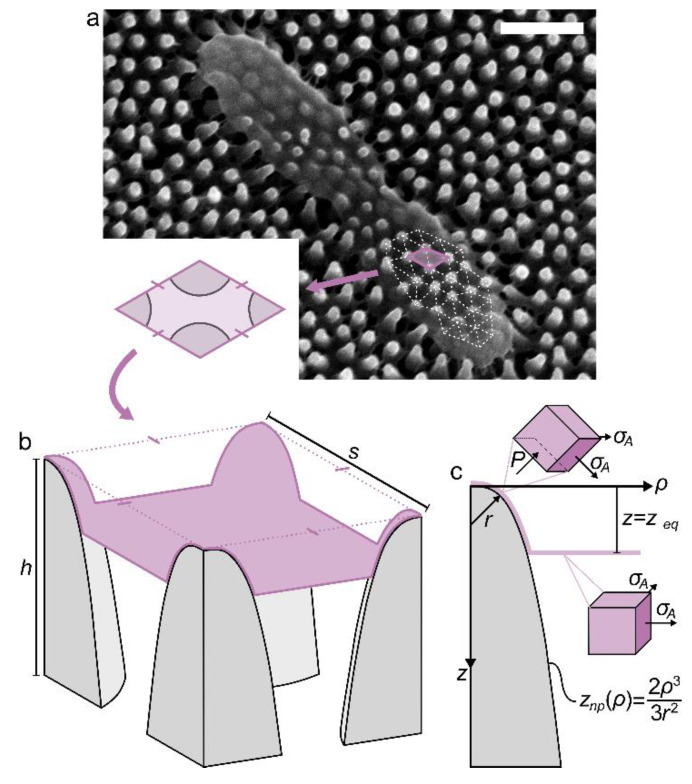
Modelling bacteria-nanopattern interaction by isolating a critical section. (**a**) Micrograph of a *Psuedemonas aeruginosa* bacteria on a *P. claripennis* wing, demonstrating ‘entrapment’ in the center of the cell. Scale bar is 500 nm; (**b**) Critical section isolated through the center of four adjacent nanopillars. The section has an edge length equivalent to the center spacing between nanopillars, *s*, and an initial area, *A*_0_. Each edge of *A*_0_ can only move in a vertical plane which is coincident with that edge. The nanopillars have a height, *h*; (**c**) Cross-section of a single nanopillar with an adhered envelope. Nanopillars are described by the smooth, non-piecewise function *z*_np_(*ρ*) = 2*ρ*^3^/3*r*^2^, where *r* is a quasi-tip radius. The envelope is adhered at equilibrium sinking depth, *z*_eq_. Contact and suspended regions of the cell experience different states of stress, indicated by stress elements noting in-plane biaxial tensile stress (*σ*_A_) and out-of-plane compressive contact pressure (*P*).

A nanopillar was modelled as an axisymmetric protrusion with a cross-section described by the positive branch of the cubic polynomial, *z*_np_(*ρ*) = 2*ρ*^3^/3*r*^2^, where *r* is a quasi-tip radius (Figure 1). Note that, although the nanopillars described by *z*_np_(*ρ*) are nowhere perfectly spherical, their tips have an approximate size of *r* and contain a point where both principal radii are equal to *r*, hence the prefix ‘quasi’ (Supplementary Materials A). The cross-section equation, *z*_np_(*ρ*), was thoughtfully selected on the basis that it was (i) monotonic, (ii) differentiable, with (iii) *z*_np_′(0) = 0, (iv) non-piecewise and (v) similar in shape to the ‘archetypal’ nanopillar found on the wings of the *Psaltoda claripennis* cicada (Figure S1a). Notably, condition three ensured that the equation did not produce a singularity (i.e., *z*_np_′(0)→∞) at the initial contact point between the nanopillar and the bacteria, and condition four allowed for more convenient parametric analysis by avoiding any cumbersome junction points. It was useful to define the inverse function of the cross-section—that is,

(1) $$\rho_{\mathrm{np}}(z)={\left(\frac{3r^{2}z}{2}\right)}^{1/3}\;\mathrm{for}\;0\leq z<h$$

where *h* is the height of the nanopillar. For any arbitrary sinking depth along the nanopillar, *z*, the nanopillar’s surface area, *A*_np_(*z*), and principal radii, *r*_1_(*z*) and *r*_2_(*z*), could be conveniently written as,

(2) $$A_{\mathrm{np}}(z)=\int 2\pi \rho_{\mathrm{np}}(z)\sqrt{1+{\rho^{\prime }}_{\mathrm{np}}{\left(z\right)}^{2}}\;dz$$

(3) $$r_{1}(z)=\rho_{\mathrm{np}}(z)\sqrt{1+{\rho^{\prime }}_{\mathrm{np}}{\left(z\right)}^{2}}$$

(4) $$r_{2}(z)=-\frac{\sqrt{{\left[1+{\rho^{\prime }}_{\mathrm{np}}{\left(z\right)}^{2}\right]}^{3}}}{{\rho^{\prime \prime }}_{\mathrm{np}}(z)}$$

defined over the same range as Equation (1).

Lastly, the nanopillars were assumed to be rigid (i.e., infinitely stiff), and thus did not deform (e.g., bend) in response to bacterial interaction. In practical terms, the elastic modulus of the nanopattern material should be at least a few orders of magnitude larger than the elastic modulus of the envelope (i.e., 10–100 MPa [32,33]). Amongst some of the ‘softest’ bactericidal nanopatterns are those composed of insect cuticle [3], polymethyl methacrylate [20], and polyethylene terephthalate [22], to name a few. These materials all have an elastic modulus on the order of 1–10 GPa [34,35,36], which likely represents the minimum requirement for sufficient structural rigidity.

### 2.2. Geometry and Properties of a Gram-Negative Bacteria

A Gram-negative bacterium was selected as the model microorganism, due to its well-known susceptibility to nanopatterned surfaces [37]. Only its envelope was modelled, because (i) the intermolecular or interparticle forces which dictate bacteria-surface interaction decay from separation and thus originate predominantly from the outermost part of the cell (i.e., the envelope) and (ii) the envelope—specifically the outer membrane-cell wall complex—is the primary load bearing component in Gram-negative bacteria [38,39,40]. The more deeply intracellular components (including the inner membrane) will simply be ‘along for the ride’ during deformation, floating freely within the cytoplasm/periplasm [41], and thus should not contribute strain energy toward the equilibrium shape. The envelope was modelled as an isotropic, two-dimensional surface having only areal stiffness, *K*_A_, and bending rigidity, *κ*. This simplified mechanical model was deemed to be a suitable approximation because the thickness of the envelope could, at times, be several times larger than the characteristic dimensions of the nanopattern. The same approximation can also be found applied in other similar scenarios of cellular adhesion [24,26,42]. The areal stiffness and bending rigidity designated to the envelope were the sum of the outer membrane and cell wall mechanical properties, given that phospholipids are permitted tangential sliding [43,44]. The outer membrane and cell wall have a wide range reported in the literature (e.g., outer membrane stiffness between 30 and 250 mN/m, and cell wall stiffness between 60 and 300 mN/m [44]), depending on probing method, experimental conditions and species composition. The present modelling considered the convenient scenario where both the outer membrane and cell wall had equivalent stretching moduli of 100 mN/m. This gave *K*_A_ = 200 mN/m and *κ* = 25 × 10^−20^ J for the combined envelope (Supplementary Materials B). Lastly, as the radius of a bacterial cell is several times larger than the typical spacing between nanopillars, its curvature will be mathematically negligible. Hence, the envelope was represented as a flat plane.

### 2.3. Boundary Conditions of the Bacteria-Nanopattern System

Model simplification was invoked to reduce the problem from the scale of the whole cell, down to a smaller, critical section (Figure 1a,b). This simplification relied on the smallness and symmetry of the nanopattern. More specifically, if the ordered nanopillar spacing was sufficiently small, the envelope area between one or more sets of four nanopillars, would effectively become ‘entrapped’ (Figure 1b). The ‘entrapped’ area, *A*_0_, can be expressed in terms of the nanopillar spacing, *s*, as

(5) A0={32s2if hexagonally ordereds2if square ordered

depending on the ordering (i.e., hex or square) of the nanopattern. For ‘entrapment’ to take place, a section between four nanopillars (Figure 1b) must be encircled by at least one layer of similar such sections. In this case, it is unlikely that material from elsewhere on the cell will pass to the ‘entrapped’ section (or sections). Thus, each edge of *A*_0_ will only move in a vertical plane that is coincident with that edge (i.e., a symmetry boundary condition) (Figure 1b). Most stringently, this demands at least three sections (*n* = 3), along the shortest axis of the cell (i.e., its diameter or width). In mathematical terms, the simplification will only be valid for nanopatterns with a spacing less than the value *s*_lim_, defined as

(6) $$s_{\lim}=\{\begin{array}{cc} \frac{d}{n}\times \frac{2}{\sqrt{3}} & \mathrm{if}\;\mathrm{hexagonally}\;\mathrm{ordered}\\ \frac{d}{n} & \mathrm{if}\;\mathrm{square}\;\mathrm{ordered} \end{array}$$

where *n* is the number of sections, and *d* is the diameter of a bacteria. As can be seen, for a hex array, the maximum valid spacing is larger (by a factor 2/√3) due to its tighter ‘packing’. As the diameter of a ‘typical’ bacterial cell vary between 500 and 1000 nm, the value of *s*_lim_ may also vary. Setting *n* = 3, for a hexagonal array the model will be valid for all values of center spacing below 192.5 nm, and invalid for spacings above 389.4 nm. Intermediate values will be potentially valid, depending on the precise width of the cell. Similarly, for a square array, the model is strictly valid, potentially valid and invalid for center spacings less than 166.7 nm, between 166.7 and 333.3 nm, and greater than 333.3 nm, respectively. That being said, nanopatterned surfaces with some bactericidal efficacy can evidently be made with larger spacing values, as long as the spacing is smaller than the diameter of the cell (for example, the hexagonal nanopattern with 595 nm center spacing in Dickson, et al. [20]). Nanopatterns’ whose spacing approaches the diameter of the cell may require different treatment. The value *s*_lim_ simply specifies the nanopattern spacings over which the mechanics described by this model are expected to hold true.

By capitalizing on the above simplification, one can circumvent regions such as the side or top of the cell whose boundary conditions are less clearly defined. Importantly, these regions are likely to be less critical because these they are (i) not as constrained as *A*_0_, and (ii) further away from the intermolecular interaction forces (which only have an effective range of—at most—a few 10 s of nm [45]). Several other works on bacteria-nanopattern interaction have employed a similar problem simplification [5,17,27].

### 2.4. Energy Considerations

The interaction of the envelope and nanopillars involves a combination of deformation and separation-dependent intermolecular or interparticle forces, which is easier to treat by energy minimization than force balance [46]. As the cell is neutrally buoyant in suspension and isolated from externally applied forces, the energy functional of the envelope comprises primarily two contributions: adhesion energy, related to the intermolecular forces which compel the envelope to deform and ‘wrap’ the nanopillars; and strain energy, which is accumulated in the envelope during this deformation process. These energies will exchange as the envelope sinks into the nanopattern until some equilibrium position is reached (Figure 1c). This was solved by conveniently expressing the adhesion and strain energies in terms of only sinking depth.

Regarding adhesion, the associated intermolecular or interparticle forces (e.g., Lifshitz van der Waals, electrostatic and acid-base forces) were collectively represented in thermodynamic terms by an energy density, *w*, which describes the free energy change between separation and contact (a.k.a. the ‘work of adhesion’). An adhesion energy density has been used to model intermolecular forces in several other studies of cellular adhesion to nanoscale features [42,47,48]. In this view, the energy released through adhesion is directly proportional to the contact area between the envelope and nanopillars. For the isolated envelope section shown in Figure 1b, contact is made with several nanopillar segments that total to one, hence the contact area is simply the surface area of one nanopillar. Thus, the adhesion energy at any arbitrary sinking depth, Γ(*z*) could be expressed as,

(7) $$\Gamma (z)=wA_{\mathrm{np}}(z)$$

Regarding strain, as per the previously defined constitutive relations, energy will be stored only from stretching and bending deformations. Stretching energy is accumulated as the envelope area increases from its initial, *A*_0_*,* to its perturbed state. As the edges of *A*_0_ only move within a vertical plane, the z-projected envelope area remains constant throughout sinking. Thus, the perturbed envelope area at any arbitrary sinking depth, *A*(*z*) was,

(8) $$A(z)=A_{0}+A_{\mathrm{np}}(z)-\pi \rho_{\mathrm{np}}{\left(z\right)}^{2}$$

Subsequently, the corresponding stretching energy, *U*_A_(*z*), was calculated to its lowest order as,

(9) $$U_{A}(z)=\frac{1}{2}K_{A}\frac{{\left[A(z)-A_{0}\right]}^{2}}{A_{0}}$$

In addition to stretching, the envelope must bend (over the contact area) in order to conform to the nanopillars. The corresponding energy was calculated using the classical expressions of Canham [49] or Helfrich [50], with a few minor adjustments: Gaussian and spontaneous curvature terms were omitted, due to up-down symmetry and absence of topological effects; and, the surface integral was transformed by substituting d*A*_np_ = *A*′_np_(*z*)d*z*. As a result, the bending energy at any arbitrary sinking depth, *U*_B_(*z*), could be expressed as,

(10) $$U_{B}(z)=\frac{1}{2}\kappa {\int \left[\frac{1}{r_{1}(z)}+\frac{1}{r_{2}(z)}\right]}^{2}{A^{\prime }}_{\mathrm{np}}(z)\;dz$$

The total potential energy of the envelope at any sinking depth, Π(*z*), was then simply the algebraic sum of the aforementioned adhesion and strain (i.e., stretching and bending) energies—that is,

(11) $$\Pi (z)=U_{A}(z)+U_{B}(z)-\Gamma (z)$$

By expressing all the energies as a function of only sinking depth, the resulting total potential energy functional could be straightforwardly minimized to find the envelope’s equilibrium sinking depth.

### 2.5. Envelope Stress State at Equilibrium

It is believed that nanopatterned surfaces elicit bactericidal activity by inducing mechanical stresses and strains in the envelope that cause physiological changes (e.g., oxidative stress or DNA damage) [13] or mechanical failure (e.g., yielding or rupture) [12]. At least two types of mechanical stresses will be incurred by the envelope as it sinks towards its equilibrium depth—an in-plane biaxial tensile stress and an out-of-plane compressive stress. 

First, to find the equilibrium sinking depth, *z*_eq_, Equation (11) was minimized (i.e., Π′(*z*_eq_) = 0). At this position, the strain energy required for an incremental gain in sinking depth matches the corresponding adhesion energy released (i.e., d*U*/d*z* = dΓ/d*z*), hence no further sinking occurs. In the case of several minima, the equilibrium sinking depth was always taken as the first minimum (Supplementary Materials C).

The in-plane biaxial tensile stress—or simply ‘areal stress’—at equilibrium, *σ*_A_, was then calculated as

(12) $$\sigma_{A}=\frac{K_{A}}{t}\left[\frac{A(z_{\mathrm{eq}})-A_{0}}{A_{0}}\right]$$

where *t* is the combined thickness of the cell wall and outer membrane, taken to have a value of 8 nm (i.e., 4 nm per component [32,51]). Equation (12) is simply the in-plane envelope tension (i.e., *K*_A_ multiplied by the areal strain, *A* − *A*_0_/*A*_0_), divided by thickness. This areal stress represents an averaged value over the entire surface area of the envelope section (Figure 1c).

The out-of-plane compressive stress—or simply, ‘contact pressure’—at equilibrium, was calculated as

(13) $$P_{\max}=\frac{2K_{A}}{r}\left[\frac{A(z_{\mathrm{eq}})-A_{0}}{A_{0}}\right]$$

which represents the contact pressure at the nanopillar tip. The derivation of Equation (13) is detailed in Supplementary Materials D. Briefly, however, contact pressure will be incurred by any part of the envelope that is in contact with the nanopillars (Figure 1c). The value of the contact pressure is simply the product of the in-plane envelope tension and the sum of the principal curvatures (i.e., 1/*r*_1_ + 1/*r*_2_), the latter of which varies depending on the position along the nanopillar. Equation (13) is this product evaluated at the tip of the nanopillar (specifically, *r*_1_ = *r*_2_ = *r*), where the principal curvatures—and thus contact pressure—will have the highest values (Supplementary Materials D).

The areal stress and contact pressure are two, orthogonal stresses (Figure 1c). In order to derive a single scalar representation of the envelope stress state, the von Mises failure theory was invoked. Von Mises theory purports that a ductile material will fail when the so-called ‘von Mises stress’ exceeds the uniaxial yield stress. This von Mises stress is a scalar value calculated by combining principal stresses; hence, the theory is useful to evaluate the effects of multiple stresses in different directions. Von Mises stress has been applied previously to evaluate biological and cellular materials such as soft tissue [52,53] and even cell envelopes [54]. In the present scenario, because the areal stress and contact pressure have perfectly orthogonal directions and opposite signs (i.e., they are tangential, tensile and normal, compressive, respectively) the von Mises stress reduces to an absolute sum of the two (see Supplementary Materials E for further details). In addition, this sum will have its maximum value (which is of greatest interest) at the nanopillar tip, where contact pressure is also maximum (i.e., *P*_max_). Altogether, the maximum von Mises stress in the envelope, *σ*_vmax_, was thus calculated as,

(14) $$\sigma_{\mathrm{vmax}}=\sigma_{A}+P_{\max}$$

As per the proposed mechanism of nanopatterned surface, the mechanical stresses in the envelope are expected to correlate with killing efficiency [12,17]. Accordingly, the areal stress, maximum contact pressure and maximum von Mises stress were investigated to elucidate the nanopattern design strategy to maximize killing efficiency.

### 2.6. Discontinuities 

At certain values of either quasi-tip radius, center spacing and/or height, abrupt changes, or discontinuities, could appear in the plots of envelope stress due to energetic barriers and physical restrictions. For instance, the bending energy gradient (i.e., d*U*_B_/d*z*) of the nanopillar increases as the nanopillar tip is made smaller. Thus, it is possible for sufficiently small tip radii to create a bending energy barrier that will be insurmountable by the work of adhesion. When this occurs, the equilibrium position of the envelope becomes *z*_eq_ ≈ 0, and all envelope stresses disappear. The precise quasi-tip radius at which this energy barrier is introduced, r*, depends on the envelope bending rigidity and the work of adhesion, and is independent of spacing (see Supplementary Materials C for further details). For example, when *κ* = 25 × 10^−20^ J and *w* = 20 mJ/m^2^, the critical radius was r* = 6.1 nm (Figure S2). The energy barrier was annotated in all the plots of envelope stress as *r* < r*.

Secondly, nanopillar interspacing and height can physically restrict the maximum sinking depth to a value of *z*_np_(*s*/2) or *h*, respectively. Thus, for sufficiently close-packed or short nanopillars, it was possible for the thermodynamic equilibrium sinking depth, *z*_eq_, to theoretically exceed these values. When this occurred, the envelope stresses were instead evaluated at *z*_np_(*s*/2) or *h*, whichever was attained first. These discontinuities were annotated in the plots of envelope stress as *z*_eq_ = *z*_np_(*s*/2) and *z*_eq_ = *h*.

### 2.7. Comparison to Previous Experimental Results

The modelling was also applied to calculate the areal stress, maximum contact pressure and maximum von Mises stress for specific nanopatterns whose killing efficiencies have already been evaluated in previous experimental studies. The previously evaluated trends in killing efficiencies were then compared with the calculated trends in envelope stresses to validate the optimal design strategy.

To identify suitable studies, the experimental literature was scanned for studies that (i) evaluated bacterial killing efficiency, (ii) of at least two differently sized nanopatterns with (iii) equivalent surface chemistry. To ensure the invoked experimental results were compatible with the governing equations and boundary conditions of the present modelling, a further two selection criteria were employed—namely, the nanopattern geometries investigated needed to be of a (iv) highly ordered nature, and the killing efficiency needed to pertain to a (v) Gram-negative species. Ultimately, the results of a total of eight studies were invoked: Nowlin, et al. [19], Dickson, et al. [20], Kelleher, et al. [21], Linklater, et al. [11], Bhadra, et al. [10], Hazell, et al. [22], Shahali, et al. [23] and Modaresifar, et al. [16]. Of these, only four studies (Dickson, et al. [20], Kelleher, et al. [21], Hazell, et al. [22], Shahali, et al. [23]) perfectly satisfied all five selection criteria. The others failed one of the latter two criteria—that is, Nowlin, et al. [19] evaluated killing efficiency against *Saccharomyces cerevisiae* yeast, which is not a Gram-negative bacteria; Linklater, et al. [11] and Bhadra, et al. [10] evaluated nanopatterned surfaces produced by reactive-ion etching of silicon wafer, which involve some degree of random spacing; and Modaresifar, et al. [16] evaluated killing efficiency against *Staphylococcus aureus,* which is also not a Gram-negative bacteria. These studies were, however, still included for comprehensiveness. It is worth mentioning that certain studies could not be included or were intentionally omitted. This includes Mainwaring, et al. [55] and Michalska, et al. [14], which did not clearly specify nanopillar tip radius, and Hazell, et al. [56] and Arias, et al. [4], which involved nanopattern geometries that were significantly disordered. 

The studies were incorporated by entering the geometries of the evaluated nanopatterns (i.e., nanopillar tip radii, center spacings, heights and type of ordering) into the model. Subsequently, the envelope stresses were plotted along with the reported efficiencies to compare the trends. 

### 2.8. Range of the Parametric Study

Overall, the effects of the four key geometric parameters on envelope stress were studied: nanopillar quasi-tip radius, *r*, between 0 and 152 nm; nanopillar center spacing, *s*, between 0 and 595 nm; nanopillar height, *h*, between 0 and 200 nm; and nanopillar ordering, either hexagonal or square. However, contour plots of radius and spacing were produced only up to the spacing value for which the mechanics of the model were expected to be strictly valid (i.e., ~200 nm for hexagonal arrays, Section 2.3). The effect of these geometric parameters was evaluated over a work of adhesion between 0 and 20 mJ/m^2^, based on previous measurements of bacterial adhesion to flat surfaces [57], atomic force microscopy tips [58] and nanoparticles [59]. 

### 2.9. Solution and Plotting

All calculations were performed in MATLAB (9.4.0.813654, R2018a, Natick, Massachusetts). The ‘base’ script for calculating the sinking depth and envelope stresses for any one set of conditions is provided in Supplementary Materials F. Additional ‘for loops’ for parametric analysis and code for plotting was added, however, the extended script is not included for brevity. Parametric analysis was performed by varying one parameter at a time, hence colinear data points were produced. The data were stored in a matrix and exported to MS Excel (2016, Redmond, Washington). Standard plots were generated with GraphPad Prism (version 9.0.0, La Jolla, California). Contour and surface plots, however, were produced by applying the GRIDFIT code in MATLAB, which could fit a smooth grid to the colinear data [60].

## 3. Results

The effects of nanopattern geometric parameters on equilibrium sinking depth, areal stress, maximum contact pressure, and maximum von Mises stress are presented herein. Of the various parameters, the effect of the radius-spacing combination was of greatest interest, which was conveyed using contour plots. Key trends with quasi-tip radius and center spacing were explained using the contour plots for a specific nanopattern case (i.e., hexagonal ordering, *h* = 200 nm and *w* = 20 mJ/m^2^), with qualitatively similar trends present for other cases. 

### 3.1. Equilibrium Sinking Depth

**Figure 2:**
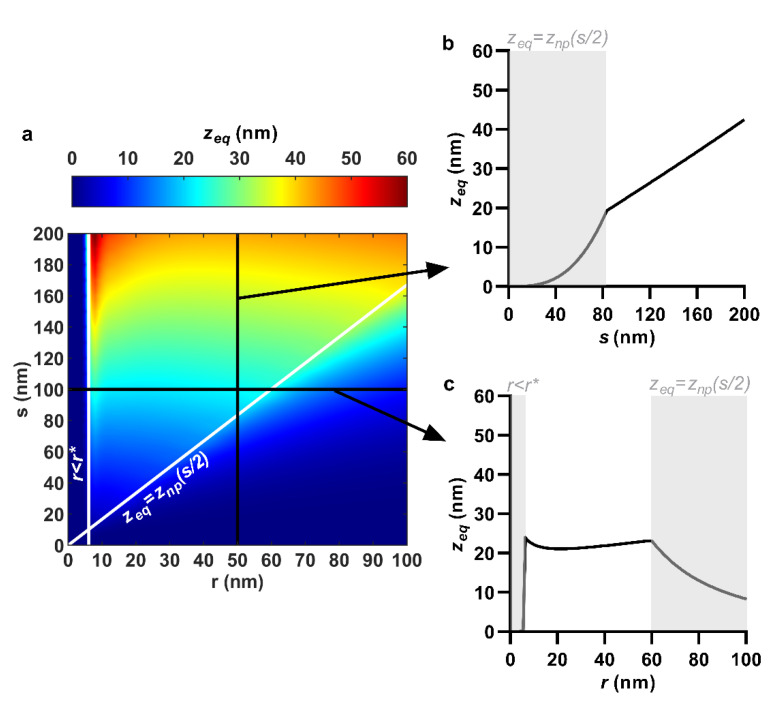
Effect of nanopillar quasi-tip radius, *r*, and center spacing, *s*, on equilibrium sinking depth, *z*_eq_. (**a**) Combined effects of quasi-tip radius and center spacing shown as a contour plot; (**b**) Selective effect of center spacing, at a fixed quasi-tip radius of 50 nm; (**c**) Selective effect of quasi-tip radius, at a fixed center spacing of 100 nm. *r* < r* and *z*_eq_ = *z*_np_(*s*/2) indicate discontinuities due to emergence of a bending energy barrier and closing of interspace, respectively. For all cases, nanopillar height, pattern ordering, and work of adhesion were 200 nm, hexagonal and 20 mJ/m^2^, respectively.

Though it is not independently related to the envelope stresses, it is worthwhile to comment on sinking depth for at least two reasons: the effects of discontinuities are most easily understood via equilibrium sinking depth, and sinking depth is one of the few variables that can be straightforwardly measured from the interaction (e.g., on tilted or cross-sectional micrographs) for potential experimental comparison. The combined effects of nanopillar quasi-tip radius and center spacing on equilibrium sinking depth are shown in Figure 2a.

The equilibrium sinking depth of the envelope was reduced by decreasing nanopillar center spacing. This predominantly occurred in a steady, linear manner; however, a more abrupt exponential decay was initiated at lower center spacing values due to the closing of interspace. For example, with quasi-tip radius fixed at 50 nm, reducing center spacing from 200 to 83.4 nm linearly reduced the equilibrium sinking depth from 42.4 to 19.3 nm (Figure 2a,b). Below a center spacing of 83.4 nm, the limited interspace between nanopillars restricted the envelope from reaching its theoretical equilibrium value. In other words, the equilibrium sinking depth simply became the depth at the midpoint between the nanopillars (i.e., *z*_eq_ = *z*_np_(*s*/2)). As this value is defined merely by the geometry of the nanopillars (i.e., *z*_np_(*s*/2) = *s*^3^/12*r*^2^), not the minimization of the envelope’s total potential energy, the form of the plots (Figure 2a–c) would change abruptly once *z*_eq_ = *z*_np_(*s*/2) was initiated. For hexagonally ordered nanopillars at an adhesion energy of 20 mJ/m^2^, this regime was initiated when the nanopillar quasi-tip radius and center spacing were combined at the ratio *r*/*s* = 0.6, shown by the diagonal white line in Figure 2a. Below this line (i.e., when *r*/*s* > 0.6), the regime *z*_eq_ = *z*_np_(*s*/2) would apply.

Quasi-tip radius, on the other hand, had a much shallower, non-monotonic effect on sinking depth. That being said, tip radii below a certain value would cause sinking depth to plumet to zero (due to the creation of an antiadhesive energy barrier) and tip radii above a certain value would drastically restrict sinking depth (due to the closing of interspace). These values were independent and dependent of center spacing, respectively. For example, with center spacing fixed at 100 nm, reducing the quasi-tip radius from 59.9 to 6.1 nm progressed the equilibrium sinking depth through a shallow valley, from a local maximum (*r* = 59.9 nm, *z*_eq_ = 23.2 nm), down to a local minimum (*r* = 21.1 nm, *z*_eq_ = 21.1 nm), and back up to a global maximum (*r* = 6.1 nm, *z*_eq_ = 24.1 nm) (Figure 2a,c). As the radius was reduced below 6.1 nm (i.e., *r* < r*), the equilibrium sinking depth suddenly became zero, due to a bending energy barrier that could not be overcome by 20 mJ/m^2^ of adhesion energy. Further increase to quasi-tip radius beyond 59.9 nm caused the sinking depth to become restricted by limited interspace (Figure 2a,c). As mentioned previously, this forced the equilibrium sinking depth to take the value *z*_np_(*s*/2), which decayed quadratically with an increasing quasi-tip radius (i.e., *z*_np_(*s*/2) = *s*^3^/12*r*^2^).

Due to the shallow effect of nanopillar quasi-tip radius, the maximum equilibrium sinking depth occurred when spacing was also maximum. In other words, the location of the sinking depth maximum was relatively insensitive to the tip radius. This can be seen, for example, from the contour plot in Figure 2a, which has a distinctly ‘vertical’ color gradient indicating that equilibrium sinking depth was mostly sensitive to center spacing. Over the range 0 < *r* < 100 nm, 0 < *s* < 200 nm, 0 < *w* < 20 mJ/m^2^, the maximum sinking depth was 60.3 nm (Figure 2a), occurring precisely at *r* = 6.1, *s* = 200 nm.

### 3.2. Areal Stress

**Figure 3:**
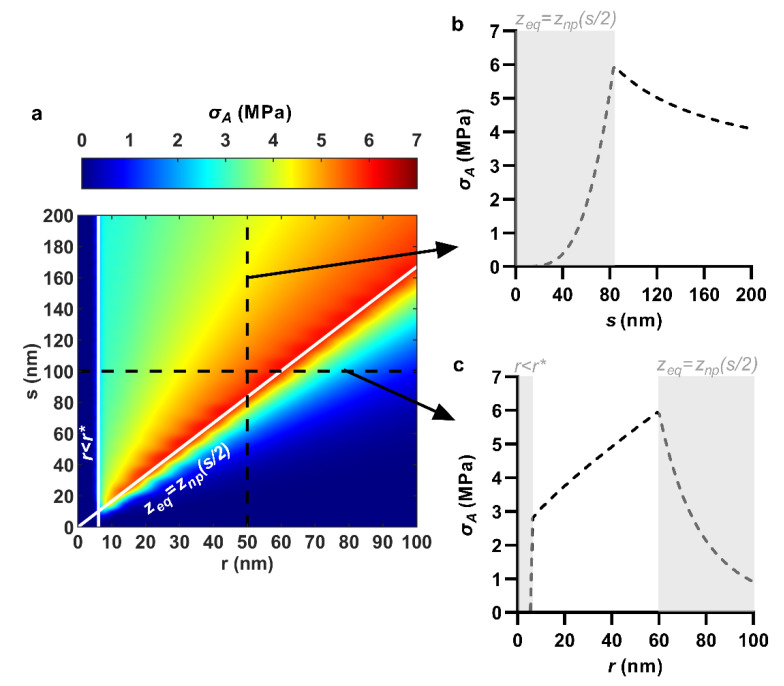
Effect of nanopillar quasi-tip radius, *r*, and center spacing, *s*, on envelope areal stress, *σ*_A_. (**a**) Combined effects of quasi-tip radius and center spacing shown as a contour plot; (**b**) Selective effect of center spacing, at a fixed quasi-tip radius of 50 nm; (**c**) Selective effect of quasi-tip radius, at a fixed center spacing of 100 nm. *r* < r* and *z*_eq_ = *z*_np_(*s*/2) indicate discontinuities due to emergence of a bending energy barrier and closing of interspace, respectively. For all cases, nanopillar height, pattern ordering, and work of adhesion were 200 nm, hexagonal and 20 mJ/m^2^, respectively.

The areal stress in the envelope was enhanced by reducing spacing and increasing tip radius. For example, when the nanopillar quasi-tip radius was fixed at 50 nm, selectively reducing the center spacing from 200 to 83.4 nm increased the areal stress at a rising rate from 4.1 to 6.0 MPa (Figure 3a,b). Similarly, when nanopillar center spacing was fixed at 100 nm, increasing the quasi-tip radius from 6.1 to 59.9 nm linearly increased the areal stress in the envelope from 2.8 to 6.0 MPa (Figure 3a,c). However, the yields in areal stress with spacing reduction and radius increase could only continue so far. Once sinking depth became restricted by limited interspace (i.e., *z*_eq_ = *z*_np_(*s*/2)), further spacing reduction or radius increase caused negative returns. As a result, the maximum areal stress was observed at the commencement of *z*_eq_ = *z*_np_(*s*/2). Recall, for the hexagonally ordered nanopillars at a work of adhesion of 20 mJ/m^2^, this occurred at the packing ratio *r/s* = 0.6. This packing ratio appears on the contour plot as the diagonal white line (Figure 3a). The value of the areal stress anywhere along this line was approximately 6 PMa, which was the maximum for the entire range. In other words, the maximum areal stress was relatively insensitive to the specific values of the tip radius and center spacing, as long as they were combined at the critical ratio. For instance, the combinations *r* = 30 nm and *s* = 50 nm, *r* = 60 nm and *s* = 100 nm, *r* = 90 nm and *s* = 150 nm and *r* = 120 nm and *s* = 200 nm all yielded between 5.91 and 6.00 MPa of areal stress. These nanopatterns are all packed tightly at the ratio *r*/*s* = 0.6.

The mechanics of these trends can be understood by considering the various governing equations in the model. The value of areal stress is determined by the final (perturbed) and initial (unperturbed) areas of the envelope. When the nanopillar center spacing is selectively reduced, so too is the initial area, *A*_0_ (see Equation (5)). This results in stretching being distributed over a smaller area, yielding a higher areal strain and stress in the envelope. On the other hand, when the nanopillar tip radius is selectively increased, there is an increase in the total surface area of the nanopattern and thus also the perturbed envelope area, *A*. Thus, albeit by slightly different routes, both the nanopillar quasi-tip radius and center spacing effectively control the in-plane stress through area. This is also likely the reason why a maximum value of areal stress can be achieved by essentially any tip radius or center spacing, as long as the other is controlled to achieve an optimally tightly packed nanopattern. In this case, ‘optimally’ signifies that the packing ratio, *r*/*s*, should only be increased until the point the sinking depth becomes physically restricted. 

### 3.3. Maximum Contact Pressure

**Figure 4:**
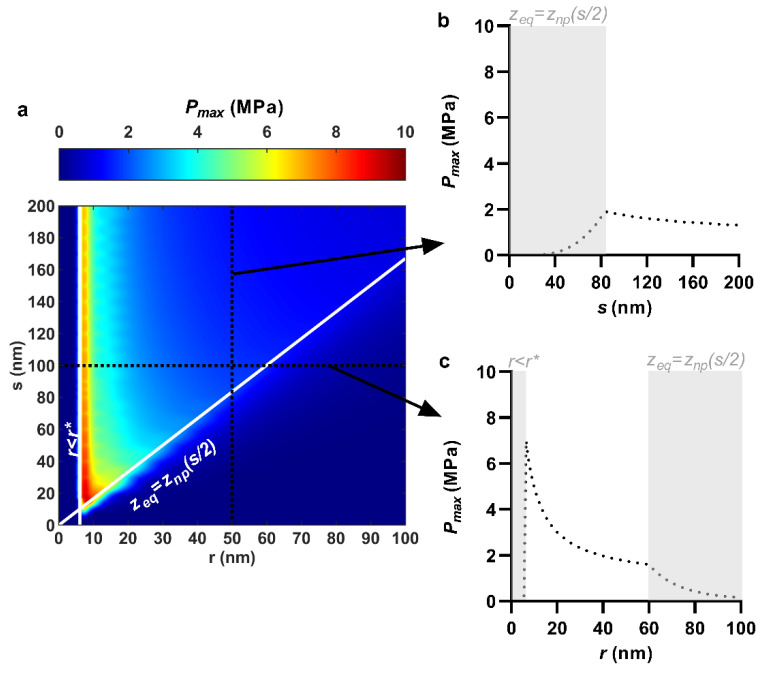
Effect of nanopillar quasi-tip radius, *r*, and center spacing, *s*, on maximum contact pressure, *P*_max_. (**a**) Combined effects of quasi-tip radius and center spacing shown as a contour plot; (**b**) Selective effect of center spacing, at a fixed quasi-tip radius of 50 nm; (**c**) Selective effect of quasi-tip radius, at a fixed center spacing of 100 nm. *r* < r* and *z*_eq_ = *z*_np_(*s*/2) indicate discontinuities due to emergence of a bending energy barrier and closing of interspace, respectively. For all cases, nanopillar height, pattern ordering, and work of adhesion were 200 nm, hexagonal and 20 mJ/m^2^, respectively.

The maximum contact pressure was enhanced predominantly by reducing the nanopillar tip radius, though also somewhat by reducing nanopillar spacing. For instance, when nanopillar quasi-tip radius was fixed at 50 nm, reducing center spacing from 200 to 83.4 nm increased the maximum contact pressure by merely 0.6 MPa, from 1.3 to 1.9 MPa (Figure 4a,b). On the other hand, when nanopillar center spacing was fixed at 100 nm, reducing the quasi-tip radius from 59.9 to 6.1 nm exponentially increased the maximum contact pressure by 5.6 MPa, from 1.6 to 7.2 MPa (Figure 4a,c). This is also conveyed visually by the distinctly ‘horizontal’ color gradient of the contour plot (Figure 4a) which indicates that the maximum contact pressure was mostly sensitive to the tip radius. The yields in maximum contact pressure by reducing tip radius (i.e., the most significant yields) could only continue until immediately before the creation of an antiadhesive bending energy barrier. For an adhesion energy of 20 mJ/m^2^ and an envelope bending rigidity of 25 × 10^−20^ J, a bending energy barrier occurred for any nanopillar quasi-tip radius below 6.1 nm (i.e., r* = 6.1 nm), irrespective of the center spacing value (Figure 4a,c). Below this value, the bending energy barrier would prevent the nanopillars from imparting contact pressure due to the elimination of sinking depth and thus envelope tension. As a result, for any one value of nanopillar center spacing, the largest maximum contact pressure always occurred at precisely *r* = r*. Moreover, as center spacing was only weakly involved, the maximum contact pressures along the line *r* = r* were similar. For instance, with *r* = 6.1 nm, the maximum contact pressure varied only between 6.9 and 7.8 MPa as center spacing was reduced from 200 to 50 nm (Figure 4a). As a result, the line *r* = r* essentially represented the largest maximum contact pressure over the entire range. This is similar to how the line *r*/*s* = 0.6 represented the maximum of the areal stress previously. The trend of increasing maximum contact pressure with decreasing tip radius follows straightforwardly from the calculation of the maximum contact, which is a product involving the nanopillar tip curvature (i.e., 1/*r*) (Supplementary Materials D). 

### 3.4. Maximum Von Mises Stress

**Figure 5:**
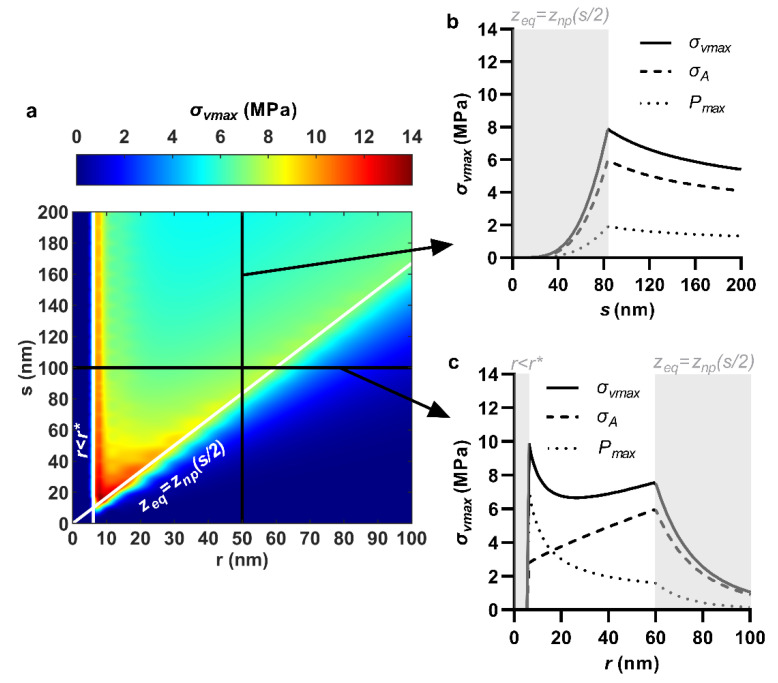
Effect of nanopillar quasi-tip radius, *r*, and center spacing, *s*, on maximum von Mises stress, *σ*_vmax_. (**a**) Combined effects of quasi-tip radius and center spacing shown as a contour plot; (**b**) Selective effect of center spacing, at a fixed quasi-tip radius of 50 nm; (**c**) Selective effect of quasi-tip radius, at a fixed center spacing of 100 nm. *r* < r* and *z*_eq_ = *z*_np_(*s*/2) indicate discontinuities due to emergence of a bending energy barrier and closing of interspace, respectively. For all cases, nanopillar height, pattern ordering, and work of adhesion were 200 nm, hexagonal and 20 mJ/m^2^, respectively.

Selective reduction in nanopillar center spacing monotonically increased the maximum von Mises stress. This was because areal stress and maximum contact pressure had commensurate trends with center spacing (Figure 5a,b), and the maximum von Mises stress was their absolute sum. Nanopillar quasi-tip radius produced a non-monotonic effect on maximum von Mises stress, due to the areal stress and contact pressure having opposite trends with quasi-tip radius (Figure 5a,c). A case for the paired modulation of both nanopillar center spacing and quasi-tip radius is also shown in Supplementary Materials G. The maximum von Mises stress had its highest values near the intersection between the lines along which its constituents were maximum—that is, near the intersection of the commencement of the two discontinuous regimes, *r* < r* and *z*_eq_ = *z*_np_(*s*/2). For example, for hexagonal ordered nanopillars at an adhesion energy of 20 mJ/m^2^, the maximum von Mises stress was 13.5 MPa, occurring at *r* = 9 nm and *s* = 15 nm, which appeared near the intersection of *r* = 6.1 nm (maximized contact pressure) and *r*/*s* = 0.6 (maximized areal stress) (Figure 5a). This type of nanopattern combines small nanopillar tip radii with tight packing to simultaneously maximize both the areal stress and the maximum contact pressure and thus consequently also their sum—the maximum von Mises stress. However, care should be taken to avoid packing nanopillars tighter than *r*/*s* = 0.6 (see Supplementary Materials H).

### 3.5. Effects of Nanopillar Height and Pattern Ordering

The effects of nanopillar height and pattern ordering were relatively straightforward and comparatively less significant, hence are shown in Supplementary Materials I. Firstly, nanopillar height only affected the equilibrium sinking depth and envelope stresses when the height was too short to suspend the sinking of the envelope. For example, with *r* = 50 nm, *s* = 100 nm and *w* = 20 mJ/m^2^, nanopillars shorter than 22.5 nm impeded the equilibrium sinking depth, and thus also the envelope stresses (Figure S4a). Above this value, however, nanopillar height had no significant effect. Over the range 0 < *r* < 100 nm, 0 < *s* < 200 nm, 0 < *w* < 20 mJ/m^2^, the maximum sinking depth was 60 nm (Figure 2a), hence it may be advisable to design nanopillar taller than this value. Secondly, all else constant, nanopatterns having a square ordering always produced marginally larger sinking depths and marginally lower envelope stresses than those with hexagonal ordering. For example, with *r* = 50 nm, *s* = 100 nm and *w* = 20 mJ/m^2^, a hexagonal ordering produced an equilibrium sinking depth, areal stress, maximum contact pressure and maximum von Mises stress of 22.5 nm, 5.5 MPa, 1.7 MPa and 7.2 MPa, whereas a square ordering produced 23.9 nm, 5.3 MPa, 1.7 MPa and 7.0 MPa, respectively (Figure S6b–e).

### 3.6. Comparison to Previous Experimental Results

**Figure 6:**
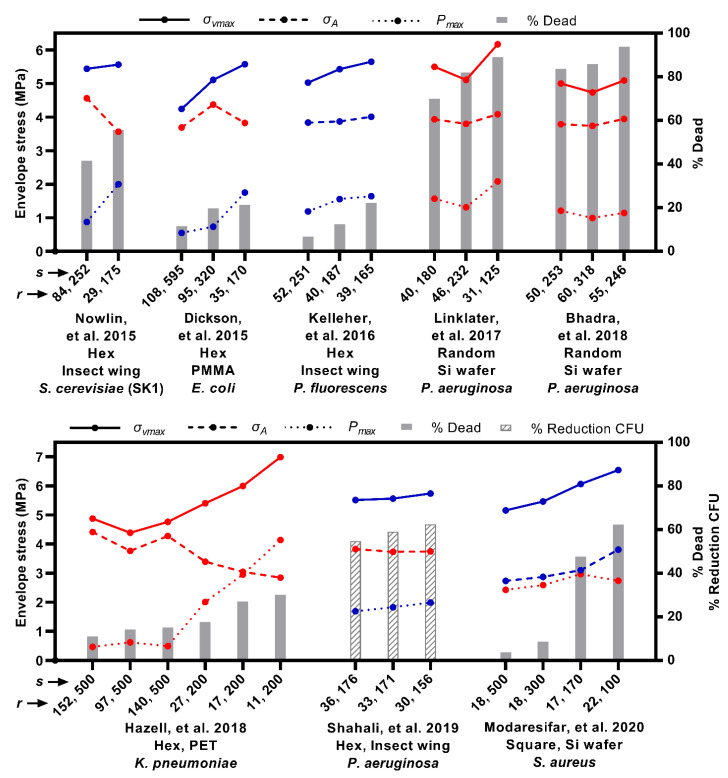
Comparison with previous experimental results to illustrate correlation between stress metrics and killing efficiency. Text in *x*-axis label cites the referenced study, and includes a description of the nanopattern ordering, material, and tested species in the study. Numbers in *x*-axis label pertain to the tip radius, *r*, and center spacing, *s*, of the nanopatterns investigated in the study. Right *y*-axis and bars plot the reported killing efficiency in terms of % dead or % reduction CFU, arranged in increasing order. Left *y*-axis and connected points plot the calculated areal stress (dashed line, *σ*_A_), maximum contact pressure (dotted line, *P*_max_) and maximum von Mises stress (solid line, *σ*_vmax_) for each of the nanopatterns (i.e., the indicated *r* and *s* values were entered into the model). A blue colored line highlights when the trends in the calculated stress and the reported killing efficiency agreed qualitatively (i.e., both consistently increased). A red colored line indicates disagreement. All calculated trends were demonstrated for the case *K*_A_ = 200 mN/m, *κ* = 25 × 10^−^^20^ J and *w* = 20 mJ/m^2^.

Figure 6 shows (i) the killing efficiency of various nanopatterns evaluated in previous experimental studies (indicated by bars), overlayed with (ii) the envelope stresses calculated in the model for each of these nanopatterns (indicated by solid, dashed, and dotted lines). The trends in killing efficiency correlated best with the theoretical maximum von Mises stress. More specifically, the trends in maximum von Mises stress qualitatively matched with the trends in killing efficiency for five out of the eight studies investigated, whereas the areal stress and maximum contact pressure matched with only two and four studies, respectively. The key observations can be summarized by elaborating on only a few of the results. For instance, Dickson, et al. [20] demonstrated increasing killing efficiency on three polymethyl methacrylate (PMMA) nanopatterns in the order *r* = 108 nm and *s* = 595 nm, *r* = 95 nm and *s* = 320 nm and *r* = 35 nm and *s* = 170 nm. Inserting these values into the model, it was shown that the nanopatterns progressively increased the maximum von Mises stress which agreed with the trend in killing efficiency (Figure 6, Dickson). It is important to note that the areal stress alone could not explain the trend. In particular, between the nanopatterns *r* = 95 nm and *s* = 320 nm and *r* = 35 nm and *s* = 170 nm, there was a reduction in areal stress, due to the reduction in the packing ratio, *r*/*s*. This reduction in areal stress was, however, less than the increase in contact pressure produced by *r* = 35 nm. Thus, despite less areal stress, there was an increase in the overall stress state of the envelope, as demonstrated by the combined von Mises stress. A similar effect was observed with the results of Nowlin, et al. [19] (Figure 6, Nowlin) and—albeit more subtly—Shahali, et al. [23] (Figure 6, Shahali). This suggested that the contact pressure must also factor into the killing efficiency. That being said, areal stress or strain should not be dismissed, as shown by the comparison with Modaresifar, et al. [16]. In Modaresifar, et al. [16], the killing efficiency against *S. aureus* was demonstrated to increase as the center spacing between nanopillars was selectively reduced from 500 to 100 nm, with the tip radius maintained at approximately *r* = 20 nm (Figure 6, Modaresifar). This trend could not be explained by the contact pressure alone, which remained approximately constant over the tested range (Figure 6, Modaresifar) (recall, contact pressure is relatively insensitive to changes in center spacing). The areal stress, however, consistently increased, in qualitative agreement with the killing efficiency, due to the increase in the packing ratio *r/s* (Figure 6, Modaresifar). As a result, the combined maximum von Mises stress also increased. Together, the results of Dickson, et al. [20] and Modaresifar, et al. [16] implied that an interplay of areal stress and contact pressure determined the killing efficiency, which could be most consistently explained by the combined von Mises stress.

That being said, the trend in the maximum von Mises stress did not totally match with the killing efficiency trends reported by Linklater, et al. [11], Bhadra, et al. [10] and—to some extent—Hazell, et al. [22]. In the case of Hazell, et al. [22], however, this was due to merely one of the six data points (i.e., *r* = 152 nm and *s* = 500 nm) (Figure 6, Hazell). This data point also involved a large value of spacing (i.e., 500 nm) which fell outside of the model’s valid range (Section 2.3), which may be cause for mismatch. For Linklater, et al. [11] and Bhadra, et al. [10] the discrepancy could have been due to non-uniform spacing. Their nanopatterns were produced by a silicon etching process in which the spacing was ultimately created by random ‘self-masking’ of adjacent nanopillars [14]. The resulting random spacing may be eliciting critical localized stress concentrations (e.g., at a random instance of two very closely spaced nanopillars) that are not accurately captured by the average value of spacing.

It should also be pointed out that the trends in the calculated envelope stress (solid, dashed, and dotted lines, Figure 6) were simply illustrated for a single case of a broadly representative model envelope (*K*_A_ = 200 mN/m and *κ* = 25 × 10^−20^ J) at a high adhesion energy (*w* = 20 mJ/m^2^). Differences in these parameters would certainly produce different calculated stresses, and thus killing efficiencies. There are probably such differences which underpin the variation in killing efficiency reported across (as opposed to within) studies. For example, Nowlin, et al. [19] and Dickson, et al. [20] reported killing efficiencies different by over 30% for two nanopatterns (*r* = 29 nm, *s* = 175 nm and *r* = 35 nm, *s* = 170 nm, respectively, Figure 6) which were quite geometrically similar. These studies involved different cell species and nanopattern compositions, for which neither the mechanical properties (e.g., *K*_A_ and *κ*) nor the interaction affinity (e.g., *w*) would be equivalent. However, it is challenging to incorporate case-specific values for *K*_A_, *κ* and *w*, because such values (i) are not broadly available and (ii) tend to be highly variable. For instance, species-specific factors such as the membrane’s phospholipid composition and the cell wall’s cross-linking degree, glycan length and pore size are known to produce variable mechanical properties across species [61,62]. However, to the best of our knowledge, only a few cell species, such as *E. coli* [63], *P. aeruginosa* [32], *S. cerevisiae* [64], *Bacillus subtilis* [65] and *Saccharopolyspora erythraea* [66] have been mechanically probed. Moreover, even for the same membrane or cell wall species, variations up to one magnitude difference have been reported in moduli and rupture values depending on strain rate [67], strained area [68], experimental configuration [69] and growth phase [70], to name a few factors. Similarly, the interaction affinity towards a nanopattern depends on a host of physicochemical properties (e.g., surface hydrophobicity and zeta potential) of the bacteria and the surface, as well as the ionic strength and pH of the suspension fluid [71,72]. Fortunately, case-specific values of *K*_A_, *κ* and *w*, do not seem to be required to understand the relationship between nanopattern geometry and stress delivery. For any one combination of *K*_A_*, κ* and *w*, the same relative trends (i.e., increasing or decreasing stress) were always observed between the different nanopattern designs within any of the studies. *K*_A_*, κ* and *w* only affected the absolute calculated stresses, whilst the relationship between nanopattern geometry and stress delivery remained the same. This meant that a design strategy to enhance envelope stress and killing efficiency through geometry could be recommended based on any one set of *K*_A_, *κ* and *w*. In practical terms, a nanopattern with a smaller tip radius and higher packing ratio will always deliver greater stresses to a cell envelope than one which comprises large, loosely packed nanopillars. Lastly, because none of the nanopillars from the invoked studies were shorter than the equilibrium sinking depth, the nanopillar heights did not factor into the envelope stress calculations.

## 4. Discussion

The results of the present modelling highlight that different stresses can be enhanced by different design strategies: packing nanopillars more tightly (i.e., increasing *r*/*s*) to increase in-plane areal stress (Figure 3); and, decreasing the nanopillar tip radius, irrespective of spacing, to increase contact pressure (Figure 4). These strategies can be combined, for instance by using tightly packed nanopillars of a small tip radius, which can simultaneously elicit high areal stresses and high contact pressures, as conveyed by the combined von Mises stress (Figure 5). Based on comparisons with previous experimental results, it is this combination which best translates to increased killing efficiency (Figure 6).

Increasing nanopillar packing (i.e., increasing *r*/*s*) has indeed been recommended in other biophysical modelling studies, though on its own this strategy is arguably problematic. Li [24], Li and Chen [25], and Xiao, et al. [26], to name a few, have all suggested this design strategy. These authors implemented a similar energy functional—involving Helfrich curvature-elasticity and thermodynamic adhesion energy—to study the interaction of bacteria, in their case, on spherically-capped cylindrical nanopillars. In Li [24], it was argued that nanopatterns with larger nanopillar radii and higher nanopillar density (smaller center spacing), would yield the greatest killing efficiency by enhancing areal strain (different to areal stress only by a constant of proportionality, *K*_A_/*t*). Similarly, Li and Chen [25] found that over the range 0 ≤ *r* ≤ 50 nm, 100 ≤ *s* ≤ 250 nm, maximum areal strain on the envelope was induced by combining the largest radius (i.e., *r* = 50 nm) with the smallest center spacing (i.e., *s* = 100 nm). In fact, the contour plots of areal strain reported by Li and Chen [25] qualitatively mirrored the areal stress contours in the present work (Figure 3a). The only difference being that Li and Chen [25] investigated over a smaller range for which interspace could not become ‘closed’—that is, spacing was always at least twice the radius. Accordingly, they concluded that areal strain was maximized at *r*/*s* = 0.5, whereas the present study—which investigated a broader range—showed that this ratio could be maximized slightly further (i.e., *r*/*s* = 0.6) without restricting sinking depth. Lastly, for spherically-capped cylindrical nanopillars, Xiao, et al. [26] also described that envelope areal strain, and presumably killing efficiency, could be enhanced by increasing nanopillar radius and increasing nanopillar density (decreasing center spacing). However, all of these studies only considered averaged areal strain or, more generally, the in-plane effects. As shown in the present work, this does not represent the total stress state of the envelope, due to the presence of a non-negligible contact pressure. By neglecting any out-of-plane effects, Li [24], Li and Chen [25], and Xiao, et al. [26] were led to the conclusion that killing efficiency should increase with increased packing ratio, *r*/*s*. Problematically, several experimental studies have demonstrated that an increased packing ratio may not always translate to increased killing efficiency—a point which is best conveyed by Nowlin, et al. [19], Dickson, et al. [20] and Hazell, et al. [22]. In Nowlin, et al. [19] and Hazell, et al. [22] it was the nanopattern with the least tight packing (least *r*/*s*) which elicited the highest bactericidal efficiency (*r* = 29 nm, *s* = 175 nm, and *r* = 11 nm, *s* = 200 nm, respectively, Figure 6) whereas in Dickson, et al. [20] it was the nanopattern with the second least (*r* = 35 nm, *s* = 170 nm, Figure 6). Importantly, these nanopatterns all had the smallest tip radii of those range investigated, implying that the absolute value of the tip radius—not just its value relative to spacing—was an important consideration. This was accounted for in the present work by including contact pressure (along with areal stress) within a combined stress (i.e., the von Mises stress) to represent the stress state of the envelope. Based on this combined stress, the recommended design strategy for enhancing envelope stress and concomitant killing efficiency was significantly different to that reported by Li [24], Li and Chen [25], and Xiao, et al. [26]. More specifically, it is shown that tight nanopillar packing must also include small tip radii in order to maximize the stress on the envelope and selectively increasing nanopillar radius is not a particularly viable strategy (Figure 6). This suggestion is more compatible with the killing efficiency trends reported by Nowlin, et al. [19], Dickson, et al. [20] and Hazell, et al. [22], whose results qualitatively agreed better with the von Mises stress, inclusive of contact pressure (Figure 6).

It is worth briefly mentioning that there are several theoretical studies such as Xue, et al. [27], Mirzaali, et al. [28] and Maleki, et al. [29] which recommend increasing nanopillar spacing to enhance envelope stress and killing efficiency—a design strategy which is not supported by the present findings. What is common in this set of theoretical studies is that they represent the interaction by a force that is independent of the surface. In the analytical elastic layer model by Xue, et al. [27], this independent force is the bacteria’s self-weight, whereas in the numerical finite element models by Mirzaali, et al. [28] and Maleki, et al. [29], it the combination of self-weight and the weight of the fluid column above the cell. The suggestion to increase spacing, therefore, stems quite straightforwardly from a greater distributed force per nanopillar, as spacing is increased. This is analogous to how a bed of nails would become more injurious if the spacing between the nails was increased. However, bacteria-surface interaction is a much more complicated problem. It is dictated (below approximately 50 nm separation) by intermolecular forces, such as London-van der Waals, electric double layer, and acid-base forces. These forces act between every site on the surface which is nearby to the cell. What this means for a nanopattern is that the net interaction of the bacteria will change depending on the number of nanopillars underneath (or near) the bacteria. In other words, a force that is independent of the interaction area should not be applied to evaluate different nanopattern designs. Although it is a smeared-out approximation, the thermodynamic adhesion energy used in the present work (as well as by Li [24], Li and Chen [25], and Xiao, et al. [26]) is at least a qualitatively correct representation of bacteria-surface interaction. By this approach, it is shown that nanopillar spacing should be reduced, a trend which agrees qualitatively with several experimental findings (Figure 6). More accurate treatment of the intermolecular forces and resulting envelope deformation (e.g., via molecular dynamics study) would be worthwhile for future studies, though it is beyond the scope of the present analytical modelling. 

In addition to providing a guide for their geometric design, the present work also offers some noteworthy perspectives on the mechanism of nanopatterned surfaces. Citing the biophysical model by Pogodin, et al. [17], it has been most commonly explained that the killing action of nanopatterned surfaces comes from an interpillar rupture of the envelope during adhesion. Problematically, rupture—when it does occur—often appears in the form of nanopillar penetration or piercing [12,14,15,73,74]. This is also seen quite plainly in the micrograph provided in Figure 1. The mismatch may be due, in part, to previous studies overlooking out-of-plane effects. Contact pressure at the nanopillar tip will indeed be significant (Figure 4), particularly for the nanopillar size range reported to be most efficiently bactericidal (i.e., tip diameters between 10 and 100 nm [1], or 5 < *r* < 50 nm). This contact pressure will contribute toward the tip being a more critical action site, consistent with the observations of nanopillar penetration. Other stresses, not explored in the present work, may also be present specifically around the nanopillar tip. For instance, one could also envision shearing of the envelope at this location. Further mechanotransduction study is needed in the field of antibacterial nanopatterned surfaces to understand how, and which types of, mechanical stresses bring about bacterial inactivation. Although rupture (facilitated through in-plane stress or strain) is commonly touted as the cause of death, there may be other critical physiological effects which may be triggered by other types of stresses. For instance, Jenkins, et al. [13] recently pointed out that Gram-negative species could be inactivated by nanopatterned surfaces without becoming ruptured. These authors implicated a heightened production of reactive oxygen species as the cause of cell death. The mechanical stress criteria (i.e., stress type and value) at which such effects are transduced is not precisely known; however, it seems that the ‘survivability limit’ and the ‘innate material strength’ of the cell may be two different quantities. Moreover, there is evidence to suggest that different types of mechanical stress will stimulate different mechanosensitive mechanisms in bacteria. For instance, by applying selective loading modes, Genova, et al. [18] demonstrated that octahedral shear stress, in particular, promotes the disassembly of efflux pumps and can render bacteria more susceptible to antimicrobials. Finite element modelling, to resolve the complex stress state of the envelope, could be paired with proteomic analyses, such as those in Genova, et al. [18] and Jenkins, et al. [13], to better understand the role of mechanical stress in bacteria-nanopattern inactivation. 

## 5. Conclusions

The present work demonstrated that significant out-of-plane contact pressures could be sustained by a bacterial envelope as a result of nanopattern interaction. To maximize both the in-plane stresses and the out-of-plane stresses, nanopattern designs should incorporate small tipped nanopillars at close packing. This recommendation agreed with killing enhancement trends reported in previous experimental studies involving differently sized nanopatterns. The work helps toward the design of highly bactericidal nanopatterned surfaces, though more detailed parallel experimental and modelling investigation is required to accurately establish the mechanism of stress deactivation on these surfaces. 

## Data Availability

The data presented in this study are available on request from the corresponding author.

## Supplementary Materials

The following are available online at https://www.mdpi.com/article/10.3390/nano11102472/s1.
