# Supplementary material for: Effects of Nanopillar Size and Spacing on Mechanical Perturbation and Bactericidal Killing Efficiency
Source: Nanomaterials (Basel). 2021 Sep 22;11(10):2472. doi: 10.3390/nano11102472 (PMC8540829; doi:10.3390/nano11102472)
Supplement: Supplementary file 1 [file nanomaterials-11-02472-s001.zip › nanomaterials-1393267-supplementary.pdf]

Supplementary materials for

Effects of nanopillar size and spacing on mechanical perturbation and bactericidal killing efficiency

Amar Velic[1], Alka Jaggessar[1], Tuquabo Tesfamichael[1], Zhiyong Li[1] and Prasad K. D. V. Yarlagadda[1,*]

[1] School of Mechanical, Medical and Process Engineering, Engineering Faculty, and Centre for Biomedical Technologies, Queensland University of Technology, 2 George St, Brisbane QLD 4000, Australia
* Correspondence: y.prasad@qut.edu.au; +617 3138 5167

A. Nanopillar geometry

A nanopillar was modelled as an axisymmetric protrusion with a cross-section described by the equation $z_{np}(\rho)=2\rho^3/3r^2$. In this equation, the parameter r acts as a 'quasi'-tip radius. To illustrate the meaning of 'quasi' in this context, the equation is demonstrated for three cases – r = 30nm, r = 60nm and r = 90nm, each at a height of 200nm – in Figure S1 below. The first case (r = 30nm) is also overlayed against the cross-section of the *Psaltoda claripennis* nanopillar, which is typically described as a perfectly spherical cone with a tip radius, base radius, and height of 30nm, 50nm and 200nm, respectively.

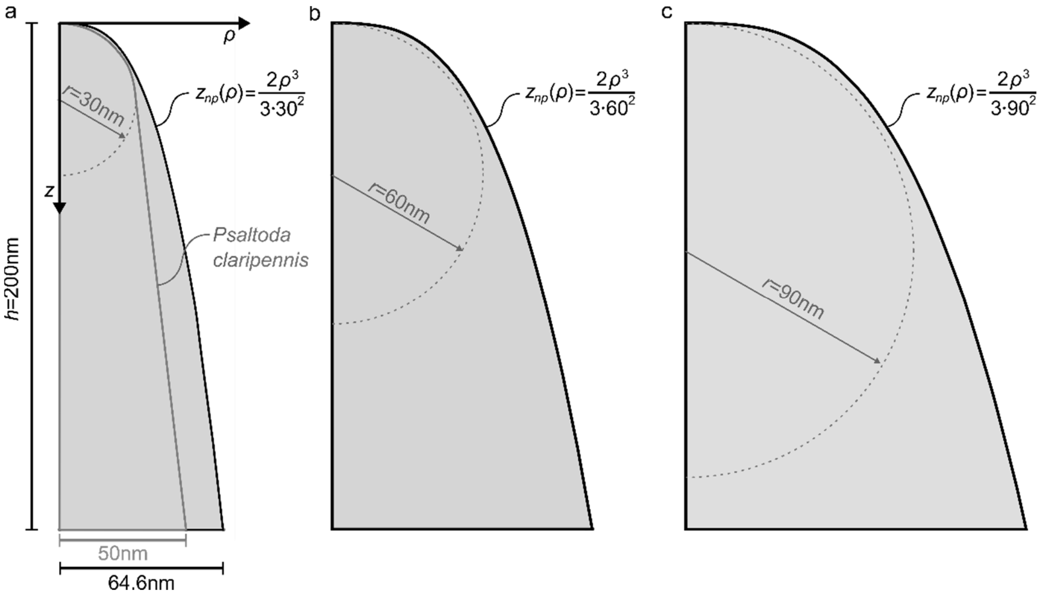

Figure S1. Cross-section of nanopillars described by the smooth, non-piecewise function $z_{np}(\rho)$ when: (a) r = 30nm, also overlayed against a *P. claripennis* nanopillar; (b) r = 60nm; (c) r = 90nm.

Two important conclusions arise from Figure S1. Firstly, for the cases r = 30nm (Figure S1a), the equation $z_{np}(\rho)$ mimics the shape of the *P. claripennis* cicada, on which the equation was modelled. Specifically, the equation closely follows this 'archetypal' cicada nanopillar until the junction point between its spherical tip and angled side wall. Though there is some additional thickness beyond this junction, it is unlikely to contribute significantly because the envelope only sinks a partial distance down the nanopillar. For instance, for a hexagonal nanopattern with r = 30, s = 180, w = 20mJ/m^2, the equilibrium sinking depth of the envelope is 38.4nm, which is only ~10nm below the junction point (26.5nm). Secondly, the parameter r roughly defines the tip size and geometry of the nanopillars described by $z_{np}(\rho)$, as can be seen in Figure S1a-c for r = 30, r = 60 and r = 90nm, respectively. Adding further to this notion, it is important to also point out that the modelled nanopillars have a position near their apex where both principal radii (r_1 and r_2) are precisely equal to r.

To demonstrate this, it is worthwhile to express the cross-section equation inversely – that is,

$$\rho_{\mathrm{np}}(z) = \left(\frac{3r^2 z}{2}\right)^{1/3} \tag{S1}$$

Subsequently, the principal radii of curvature at any depth along the nanopillar, $r_1(z)$ and $r_2(z)$, can be conveneitly expressed as,

$$r_1(z) = \rho_{\mathrm{np}}(z)\sqrt{1+\rho'_{\mathrm{np}}(z)^2} \tag{S2}$$

$$r_2(z) = -\frac{\sqrt{\left[1+\rho'_{\mathrm{np}}(z)^2\right]^3}}{\rho''_{\mathrm{np}}(z)} \tag{S3}$$

respectively. Setting $r_1(z) = r_2(z)$, one finds that at a depth of $\sqrt{2}/6r$ (~$0.23r$) both principal radii are equivalent. Substituting this value into Eq.(S2) and Eq.(S3) gives $r_1(\sqrt{2}/6r) = r_2(\sqrt{2}/6r)=r$ – that is, both principal radii have the value r. Thus, although the nanopillars described by $z_{np}(\rho)$ are not perfectly spherical, the parameter r defines the approximate size of the tip and its curvature, hence the term 'quasi'-tip radius. The key benefit of using $z_{\mathrm{np}}(\rho)$ to describe the nanopillar shape is that only one parameter (i.e., r) controls the 'width' of the nanopillars, thus junction points – which can be cumbersome in parametric analysis - are avoided.

B. Calculation of bending rigidities

The bending rigidities of the outer membrane and cell wall were calculated by invoking the polymer brush model and thin-plate theory, respectively. For the outer membrane, which is a phospholipid bilayer, the areal stiffness, K_{AOM}, and bending rigidity, κ_{OM}, are related by,

$$\kappa_{\mathrm{OM}} = K_{\mathrm{AOM}} \frac{t_{\mathrm{OM}}^{2}}{24} \quad \text{(S4)}$$

where t_{OM} is the thickness of the membrane between headgroups. The areal stiffness and thickness of the outer membrane were taken from previous reports as 100mN/m and 4nm, respectively, thus the bending rigidity calculated by Eq. (S4) was ~7×10^{-20}J. In the case of the cell wall, which is a single elastic layer of thickness t_{CW}, the relation between the areal stiffness, K_{ACW}, and the bending rigidity, κ_{OM}, is given – from thin plate theory - by,

$$\kappa_{\mathrm{CW}} = K_{\mathrm{ACW}} \frac{t_{\mathrm{CW}}^{2}}{6(1+v)} \quad \text{(S5)}$$

The areal stiffness and thickness of the cell wall were taken from previous reports as 100mN/m and 4nm, respectively, thus the bending rigidity calculated by Eq. (S5) was ~18×10^{-20}J (assuming a Poisson's ratio of $v = 0.5$).

As the phospholipids (to which the cell wall is attached via lipoproteins) are permitted to slide freely, the combined mechanical properties of the envelope will simply be the sum of the outer membrane and cell wall properties - that is K_A=200mN/m and κ=25×10^{-20}J, as found in the main text.

C. Identifying the equilibrium sinking depth

Once a bacterium touches down on the tips of the nanopillars, intermolecular or interparticle forces will pull the envelope further into contact via a propagating bond front. The bond front will continue to propagate until the point the elastic reaction forces in the envelope equal or begin to exceed the intermolecular forces. This instance will be the equilibrium position of the envelope. In the present work, this adhesion process was represented by a thermodynamic approach. Accordingly, the intermolecular or interparticle forces were expressed as an adhesion energy, Γ, and the elastic reaction forces of the envelope were expressed in terms of strain energy, *U*. In this scenario, equilibrium is defined by the rate of change of these energies. For instance, at equilibrium, the strain energy required for an incremental gain in sinking depth will be equivalent to the corresponding adhesion energy released (i.e. $dU/dz = d\Gamma/dz$). As the total potential energy of the envelope, Π, comprises only these two components (i.e., $\Pi = U - \Gamma$), the equilibrium position appears as a minimum.

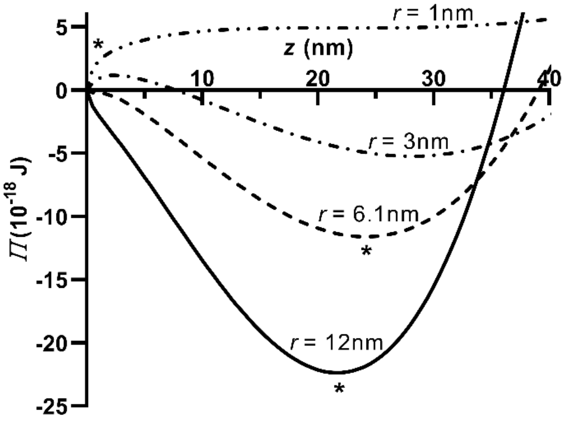

Figure S2. Total potential energy of the bacterial envelope, Π, at any sinking depth, *z*, on nanopillar with a quasi-tip radius, *r*, of 1, 3, 6.1 or 12nm. The nanopattern spacing, height and ordering are 100nm, 200nm, and hexagonal, respectively. The work of adhesion is 20mJ/m^2.

With this in mind, Figure S2 above shows the total potential energy of the envelope at any sinking depth, $\Pi(z)$, for four different values of nanopillar quasi-tip radius, *r*. In all cases, the nanopillar center spacing, *s*, nanopillar height, *h*, and work of adhesion, *w*, are 100nm, 200nm and 20mJ/m^2, respectively. The equilibrium sinking depth, z_{eq}, is thus the sinking depth corresponding to the minimum in the curve. For r = 12nm and r = 6.1nm, the plots of potential energy contain only one minimum, which becomes the equilibrium sinking depth of the envelope (i.e., 21.7nm and 24.1nm, respectively). For r = 3nm and r = 1nm, however, the potential energy initially increases from the origin, which yields an early local (r = 3nm) or global (r = 1nm) minimum at z~0nm. The physical meaning of this condition is that the rate of strain energy accumulation is initially higher than the rate of adhesion energy release. This is caused specifically by the large bending energy gradient at the nanopillar tip which cannot be surmounted by the adhesion energy (i.e. $dU_B/dz > d\Gamma/dz$). As a result, the envelope cannot sink down the nanopillar, and will remain at the first minimum (i.e. z_{eq}~0nm).

In fact, all tip radii below a critical value will produce this condition. For perfectly spherically tipped nanopillars it can be shown that this critical tip radius has a value of $\sqrt{(2\kappa/w)}$ (where κ is the bending rigidity). However, as the nanopillars described by $z_{np}(\rho)$ are not perfectly spherical, the critical quasi-tip radius is slightly larger (e.g., 6.1nm at $\kappa = 25\times10^{-20}$ and w = 20mJ/m^2). Nanopillars with any quasi-tip radius below this value will elicit z_{eq} ~ 0nm (Figure S2).

D. Derivation of contact pressure

In bacteria-nanopattern interaction, contact pressure will be generated between the envelope and nanopillars. This is due to a combination of contact, in-plane tension, and curvature. More specifically, because the envelope's in-plane tension has a tangential direction to the curved nanopillar surface, a normal force will also act on the envelope. To derive the contact pressure, the normal forces generated on an infinitesimal area of the envelope were considered.

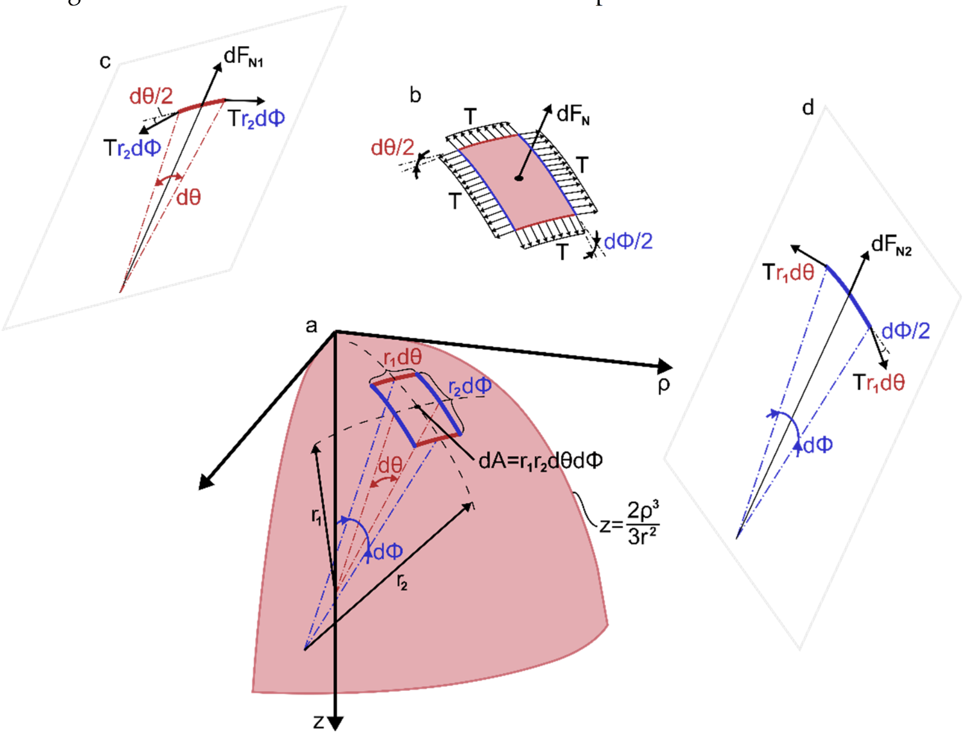

Figure S3. Contact pressure acting on the envelope. (a) An arbitrary area element, dA, on an arbitrary segment of an envelope contacting a nanopillar. r_1 and r_2 represent the principal radii of curvature at the center of the area element. dθ and dΦ are infinitesimal angles in the corresponding principal planes, expanding from the center of the area element; (b) Free body diagram of the area element, illustrating the generation of an infinitesimal normal force (dF_N) from the in-plane tension (T); (c-d) Two-dimensional projections of the previous free body diagram in the principal planes. The total infinitesimal normal force (dF_N) can be viewed as the sum of the infinitesimal normal forces (dF_{N1} and dF_{N2}) appearing in these planes.

Figure S3 shows an arbitrary infinitesimal element of area dA isolated from a segment of an envelope, wrapped around a nanopillar. For this derivation, it is most convenient to use a coordinate system whose axes coincide with the planes of principal curvature at the center of element. By this approach, the edge lengths of the element dA can be conveniently expressed as the principal radii multiplied by an infinitesimal angle change within the corresponding plane - that is r_1dθ and r_2dΦ. The area of element dA is then,

$$\mathrm{d}A = r_1 r_2 \mathrm{d}\theta \mathrm{d}\phi \tag{S6}$$

Due to the curvature of the nanopillar, the in-plane envelope tension (T) acts on the edges of the area element at a angle of depression of dθ/2 and dΦ/2 (Figure S3b). The resulting infinitesimal normal force (dF_N) on the element can then be calculated by force balance in the direction of the normal vector at the center of the area element. For clarity, the force balance can be represented within each principal plane and subsequently combined (Figure S3c,d). In the first principal plane,

$$\mathrm{d}F_{N1} = 2Tr_2 \mathrm{d}\phi \sin(\mathrm{d}\theta / 2) \tag{S7}$$

Note that the edge length r_2dΦ enters Eq.(S7) because tension is a distributed load (i.e., force per unit length). Similarly, in the second principal plane,

$$\mathrm{d}F_{N2} = 2Tr_1 \mathrm{d}\theta \sin(\mathrm{d}\phi / 2) \tag{S8}$$

The sum of these components gives the total infinitesimal normal force acting on the area element - that is,

$$\mathrm{d}F_{\mathrm{N}} = \mathrm{d}F_{\mathrm{N1}} + \mathrm{d}F_{\mathrm{N2}} \tag{S9}$$

Contact pressure, P, is simply lim $\mathrm{d}F_{\mathrm{N}}/\mathrm{d}A$ as $\mathrm{d}A\to 0$. Using Eq.(S6)-(S9), however, this can also be expressed as

$$P = \lim_{\mathrm{d}\theta,\mathrm{d}\phi\to 0} \frac{2Tr_2\mathrm{d}\phi\sin(\mathrm{d}\theta/2) + 2Tr_1\mathrm{d}\theta\sin(\mathrm{d}\phi/2)}{r_1 r_2 \mathrm{d}\theta\mathrm{d}\phi} \tag{S10}$$

The limit of Eq.(S10) is indeterminate if attempted by direct substitution, or if approached from either axis (lim $\mathrm{d}\theta\to 0$, $\mathrm{d}\Phi=0$ or lim $\mathrm{d}\Phi\to 0$, $\mathrm{d}\theta=0$). That being said, it can be easily determined if approached from any other linear direction, mathematically denoted as lim $\mathrm{d}\theta\to 0$, $\mathrm{d}\Phi = \alpha\mathrm{d}\theta$, where α is any real, non-zero number. Executing the limit in this way, it can be shown,

$$\begin{aligned}
P &= \lim_{\substack{\mathrm{d}\phi=\alpha\mathrm{d}\theta \\ \mathrm{d}\theta\to 0}} \frac{2Tr_2\mathrm{d}\phi\sin(\mathrm{d}\theta/2) + 2Tr_1\mathrm{d}\theta\sin(\mathrm{d}\phi/2)}{r_1 r_2\mathrm{d}\theta\mathrm{d}\phi}, \quad \text{where} \quad \alpha \in R \mid \alpha \neq 0 \\
&= \lim_{\substack{\mathrm{d}\phi=\alpha\mathrm{d}\theta \\ \mathrm{d}\theta\to 0}} \frac{2Tr_2\alpha\mathrm{d}\theta\sin(\mathrm{d}\theta/2) + 2Tr_1\mathrm{d}\theta\sin(\alpha\mathrm{d}\theta/2)}{r_1 r_2\mathrm{d}\theta\alpha\mathrm{d}\theta} \\
&= \lim_{\substack{\mathrm{d}\phi=\alpha\mathrm{d}\theta \\ \mathrm{d}\theta\to 0}} \frac{2T}{r_1 r_2}\left[\frac{r_2\sin(\mathrm{d}\theta/2)}{\mathrm{d}\theta} + \frac{r_1\sin(\alpha\mathrm{d}\theta/2)}{\alpha\mathrm{d}\theta}\right] \\
&= \lim_{\substack{\mathrm{d}\phi=\alpha\mathrm{d}\theta \\ \mathrm{d}\theta\to 0}} \frac{2T}{r_1 r_2}\left[\frac{r_2\sin(\mathrm{d}\theta/2)}{2(\mathrm{d}\theta/2)} + \frac{r_1\sin(\alpha\mathrm{d}\theta/2)}{2(\alpha\mathrm{d}\theta/2)}\right] \\
&= \frac{2T}{r_1 r_2}\left(\frac{r_2 + r_1}{2}\right) \\
&= T\left(\frac{1}{r_1} + \frac{1}{r_2}\right)
\end{aligned} \tag{S11}$$

As can be seen, contact pressure will simply be the product of the in-plane envelope tension (T) and sum of the principal radii ($1/r_1 + 1/r_2$) of the nanopillar. This is very similar to the expression T/r one might find in a classical mechanics textbook for the contact pressure between a belt and a cylinder (for instance, in a pulley system). The only difference being that that the nanopillar is doubly curved, therefore the contact pressure includes an additional $1/r$ multiplied by tension.

Evidently, the contact pressure will vary at different locations on the nanopillar, depending on curvatures at that location. However, the highest curvatures - and thus contact pressure – will occur at the nanopillar tip. Thus, it is most useful to evaluate the contact pressure at $r_1 = r_2 = r$, for which the bottom line of Eq. (S11) becomes $P_{\mathrm{max}} = 2T/r$. The contact pressure in the main text is included in the manner, with tension written as the areal strain at equilibrium, $A(z_{\mathrm{eq}})$-A_0/A_0, multiplied by the stretching modulus, K_{A} – that is,

$$P_{\mathrm{max}} = \frac{2K_{\mathrm{A}}}{r}\left[\frac{A(z_{\mathrm{eq}}) - A_0}{A_0}\right] \tag{S12}$$

E. Calculation of von Mises stress

The von Mises stress is the key metric used to assess failure (i.e., yielding) within the popular 'von Mises' theory. This theory purports that a ductile material will fail when the 'von Mises stress' exceeds the uniaxial yield stress. The 'von Mises stress' is calculated by combining principal stresses (σ_{11}, σ_{22}, and σ_{33}) according to Eq. (S13), resulting in a convenient, single scalar value. Accordingly, the 'von Mises stress' is useful to evaluate the combined effect of multiple stresses in different directions.

$$\sigma_{\mathrm{v}} = \sqrt{\frac{(\sigma_{11}-\sigma_{22})^2+(\sigma_{22}-\sigma_{33})^2+(\sigma_{33}-\sigma_{11})^2}{2}} \tag{S13}$$

In bacteria-nanopattern interaction, certain parts of the envelope will subject to multiaxial stresses. Specifically, the region of the envelope in contact with the envelope will be subjected to at least two, orthogonal stresses: an 'areal stress', σ_A, and a 'contact pressure', P. The former is in-plane (tangential), biaxial and tensile, whilst the latter is out-of-plane (normal), uniaxial and compressive (see Figure 1 in main text). Eq. (S13) can be used the calculate the von Mises stress in such a scenario. The principal stresses in Eq. (S13) can be replaced by the areal stress and contact pressure, respectively (i.e. $\sigma_{11} = \sigma_{22} = \sigma_A$ *and* $\sigma_{33} = P$, if directions 1 and 2 are set as the in-plane directions). As contact pressure is compressive, it must be entered as a negative in Eq. (S13). Consequently, the theoretical von Mises stress in the contact region of the envelope is,

$$\begin{aligned}\sigma_{\mathrm{v}} &= \sqrt{\frac{(\sigma_{\mathrm{A}}-\sigma_{\mathrm{A}})^2+[\sigma_{\mathrm{A}}-(-P)]^2+[(-P)-\sigma_{\mathrm{A}}]^2}{2}} \\ &= \sqrt{\frac{(\sigma_{\mathrm{A}}+P)^2+(P+\sigma_{\mathrm{A}})^2}{2}} \\ &= \sigma_{\mathrm{A}}+P\end{aligned} \tag{S14}$$

which is simply the absolute sum of areal stress and contact pressure.

As seen in the bottom line of Eq. (S11), the contact pressure, P, will vary at different locations on the nanopillar. Therefore, so too will the von Mises stress. It is worth mentioning that in the suspended region, where there is no contact, the von Mises stress will simply be the value of areal stress (i.e. P=0, therefore $\sigma_v = \sigma_A$ as). The maximum theoretical von Mises stress over the entire surface of the envelope will occur specifically at the nanopillar tip, where contact pressure also has its maximum. That is,

$$\sigma_{\mathrm{vmax}} = \sigma_{\mathrm{A}} + P_{\mathrm{max}} \tag{S15}$$

F. MATLAB script

The following is the 'base' script for calculating the sinking depth and envelope stresses for any one set of conditions (i.e., any single value of r, s, h, ordering type, and w).

Envelope material properties

```
% Outer membrane
tom=4e-9; % Outer membrane thickness (m)
smodom=0.100; % Outer membrane areal stiffness (N/m)
cmodbl=smodom*tom^2/24; % Outer mebrane bending rigidity, calculated based on
the polymer brush model (J)
% Cell wall
tcw=4e-9; % Cell wall thickness (m)
smodcw=0.100; % Cell wall areal stiffness (Pa)
cmodcw=smodcw*tcw^2/(6*(1+0.5)); % Cell wall bending rigidity, calculated
based on thin plate theory (J)
% Combined envelope
tenv=tcw+tom; % Envelope thickness (m)
smod=smodom+smodcw; % Envelope areal stiffness (N/m)
cmod=cmodbl+cmodcw; % Cell wall bending rigidity (J)
```

Nanopattern Parameters

```
r=30e-9; % Nanopillar quasi-tip radius (m)
s=180e-9; % Nanopillar center spacing (m)
h=200e-9; % Nanopillar height (m)
```

Interaction Parameters

```
w=-0.02; %Work of adhesion (J/m2)
```

Nanopillar geometry equation

```
syms z
rho=(3*r^2*z/2)^(1/3); % Nanopillar geometry equation
```

Areas

```
a0=sqrt(3)/2*s^2; % Initial envelope section area based on symmetry of a
hexagonal array
%a0=s^2; % Initial envelope section area based on symmetry of a square array
anp=real(int(2*pi*rho*sqrt(1+diff(rho)^2))); % Nanopillar surface area (or
contact area) at any sinking depth
a=a0+anp-pi*rho^2; % Total envelope area at any sinking depth
```

Stretching Energy

```
stretch=(1/2)*smod*(a-a0)^(2)/a0; % Stretching energy at any sinking depth
```

Bending energy

```
r1=rho*sqrt(1+diff(rho)^2); % Radius of first principle curvature at any
sinking depth
r2=(1+diff(rho)^(-2))^(3/2)/(-1*(diff(diff(rho)))/(diff(rho)^3)); % Radius of
second principle curvature at any sinking depth
bend=(1/2)*cmod*int((1/r1+1/r2)^2*2*pi*rho*sqrt(1+diff(rho)^2)); % Bending
energy at any sinking depth
```

Adhesion Energy

```
adhesion=w*anp; % Adhesion energy at any sinking depth
```

Calculating the equilibrium sinking depth, zeq

```
pot=stretch+bend+adhesion; % Total potential energy of the envelope at any
sinking depth
potfun=matlabFunction(pot); % As above, expressed as a Matlab function
dpot=diff(pot); % Derivative of the total potential energy of the envelope at
any sinking depth
dpotfun=matlabFunction(dpot); % As above, expressed as a Matlab function
zeq=fzero(dpotfun,[0.0000000001e-9,10000000000e-9]); % Finding the minimum of
the total potential energy
```

Envelope stresses

```
% Areal strain at equilibrium
arealstrain=(a-a0)/a0; % Areak straub at any sinking depth
   arealstrainfun=matlabFunction(arealstrain); % As above, expressed as a
Matlab function
   arealstraineq=arealstrainfun(zeq); % Areal strain at equilibrium
% Areal stress at equilibrium
tensioneq=smod*arealstraineq; % Envelope tension at equilibrium
arealstresseq=tensioneq/tenv; % Areal stress at equilibrium
% Maximum contact pressure at equilibrium
pressuremaxeq=2*tensioneq/r;
% Maximum von Mises stress at equilibrium
vmsmaxeq=arealstresseq+pressuremaxeq;
```

Results storage

```
fullstore=[r*1e9; s*1e9; zeq*1e9; arealstresseq*1e-6; pressuremaxeq*1e-6;
vmsmaxeq*1e-6]';
```

Published with MATLAB® R2018a

G. Paired modulation of radius and spacing

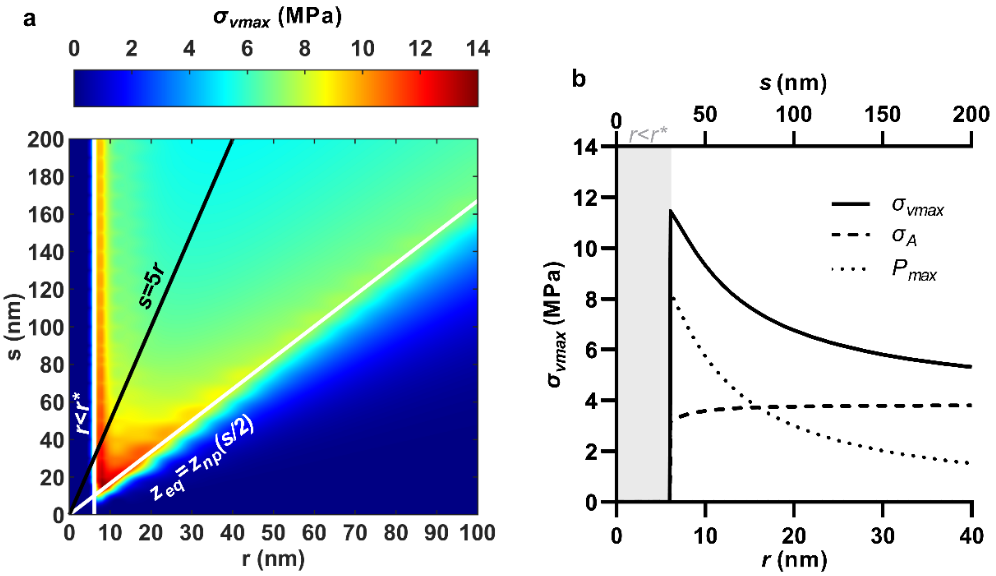

Figure S4. Effect of paired modulation of nanopillar quasi-tip radius, r, and center spacing, s, according to the ratio $s = 5r$. (a) Contour plot of maximum von Mises stress, σ_{vmax}, highlighting the line $s = 5r$; (b) Maximum von Mises stress, σ_{vmax}, areal stress, σ_A, and maximum contact pressure, P_{max}. when quasi-tip radius and spacing are changed in the ratio $s = 5r$. $r<r^*$ and $z_{eq} = z_{np}(s/2)$ indicate discontinuities due to emergence of a bending energy barrier and closing of interspace, respectively. For all cases, nanopillar height, pattern ordering, and work of adhesion were 200nm, hexagonal and 20mJ/m^2, respectively.

H. Increasing nanopattern packing

Increasing the nanopattern packing ratio (*r*/*s*) is a viable strategy to increase envelope stress and concomitant killing efficiency, however, excessively packed nanopillars will yield diminishing returns. Figure S5 below illustrates the envelope von Mises stress (Figure S5a) for four different hexagonally ordered nanopatterns ranging from low to excessive tight packing (Figure S5b-e). In this scenario, the packing ratio (*r*/*s*) is increased by selectively reducing center spacing from *s* = 200nm (Figure S5b) to *s* = 25nm (Figure S5e), whilst the quasi-tip radius maintained at *r* = 50nm. From nanopattern b (*r* = 50, *s* = 200nm) to c (*r* = 50nm, *s* = 100nm) there is an increase in the von Mises stress, which stems primarily from an increase in areal stress. However, further spacing reduction to nanopatterns d (*r* = 50nm, *s* = 50nm) and e (*r* = 50nm, *s* = 25nm), results in a sharp decrease in envelope stress. This is due to the limited sinking depth at the midpoint of the pillars (i.e., $z_{np}(s/2)$). The resulting nanopatterns with excessively high packing (Figure S5d-e) resemble near 'flat' surfaces, hence their capacity to deliver stress to the envelope is understandably low.

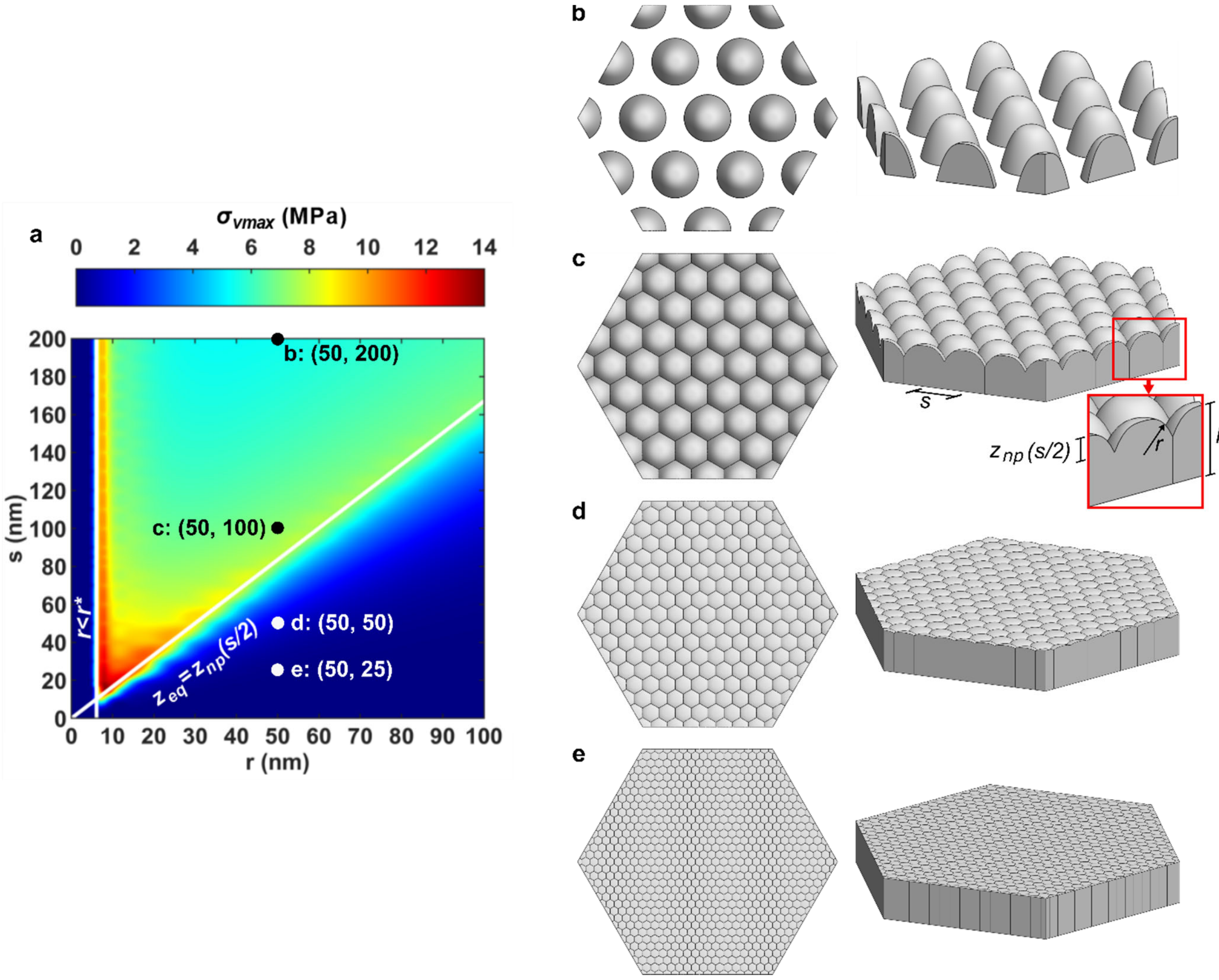

Figure S5. Increasing nanopattern packing ratio (*r*/*s*) at a constant quasi-tip radius of *r* = 50nm. (a) Contour plot of maximum von Mises stress, σ_{vmax}, for nanopatterns with hexagonal ordering and an adhesion energy of 20mJ/m^2; (b) Center spacing, *s* = 200nm; (c) *s* = 100nm, with insert highlighting the maximum sinking depth between pillars, $z_{np}(s/2)$, and nanopillar height, *h*; (d) *s* = 50nm; (e) s = 25nm. All 3D models shown with a nanopillar height of 100nm.

I. Effects of nanopillar height and pattern ordering

The nanopillar height only affected the interaction when it was insufficient to suspend the theoretical equilibrium sinking depth of envelope. In these cases, the equilibrium sinking depth of the envelope simply took the value of the nanopillar height (i.e., $z_{eq} = h$), as highlighted in Figure S6a. When this occurred, the envelope stresses would also be impeded (Figure S6a). However, once the height could suspend the equilibrium sinking, additional height had no significant effect. Secondly, all else constant, nanopatterns having a square ordering always produced marginally larger sinking depths and marginally lower envelope stresses than those with hexagonal ordering (Figure S6b-e).

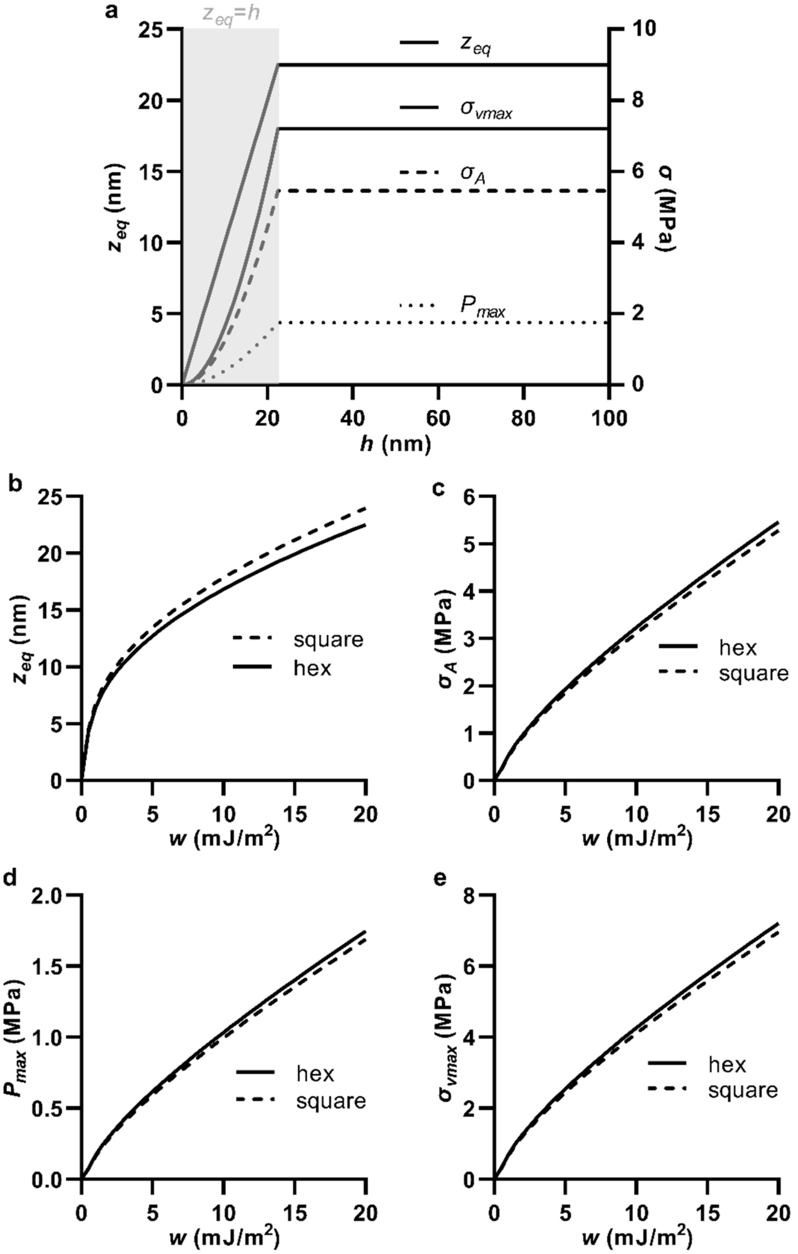

Figure S6. Effect of nanopillar height and pattern ordering on the equilibrium sinking depth, z_{eq}, areal stress, σ_A, maximum contact pressure, P_{max}, and maximum von Mises stress, σ_{vmax}. (a) Effect of height, demonstrated for a hexagonally ordered nanopattern with r = 50nm, s = 100nm and w = 20mJ/m^2; (b-e) Effect of ordering shape, demonstrated for a nanopattern with r = 50nm and s = 100nm.
